# Review on unobtrusive estimation of team processes underlying team situation awareness: shared mental models and team coordination

**DOI:** 10.3389/fnrgo.2026.1864518

**Published:** 2026-10-14

**Authors:** Emanuela Zhecheva, Eric Groen, Pim Haselager, Anne-Marie Brouwer

**Affiliations:** 1 Donders Institute for Brain, Cognition and Behaviour, Radboud University, Nijmegen, Netherlands; 2 Department of Human Performance, TNO Netherlands Organisation for Applied Scientific Research, Soesterberg, Netherlands; 3 Faculty of Engineering and Applied Sciences, Cranfield University, Bedford, United Kingdom

**Keywords:** behavior, measure, physiology, review, shared mental models, team, team coordination, team situation awareness

## Abstract

**Introduction:**

Team situation awareness (SA) is essential for effective team performance. Yet, current methods rely on potentially biased self-reports and obtrusive tools such as task probes. Objective, unobtrusive, and continuous methods for monitoring team SA could support its improvement. In contrast to individual SA, unobtrusive measures of team SA have not been systematically reviewed. Because team SA is an umbrella concept, we adapted a framework proposed by Salmon et al., identifying two components that can potentially be measured unobtrusively: shared mental models (SMMs) and team coordination. This systematic review examines behavioural and physiological technologies that may enable unobtrusive measurement of these components.

**Methods:**

We conducted a systematic literature review of studies investigating behavioural technologies, such as verbal communication and eye-tracking, and physiological technologies, such as electroencephalography, for unobtrusive measurement of team SMMs and coordination. We searched Web of Science (May and July 2025) for relevant literature published between 2000 and 2025. We included peer-reviewed articles published in English. In total, 19 studies addressing team SMMs and 33 addressing team coordination were included. We extracted sample and team size, task environment, operationalisation of team SMMs and coordination, and their relationship with unobtrusive measures.

**Results:**

Cardiac measures (42%) and verbal communication (47%) were most popular for estimating team SMMs, while functional near-infrared spectroscopy (27%) and cardiac measures (30%) were most popular for estimating team coordination. Interpersonal synchrony—the temporal contiguity and interdependence of physiological and/or behavioural activity between teammates—was applied in 70% of team coordination studies. Studies covered diverse tasks and settings, from laboratory-based cognitive tasks and controlled physical tasks to real-world environments, including surgery. Various methods were used to operationalise SMMs and coordination, including participant experience, expert assessment, and self-reports.

**Discussion:**

Findings suggest that team SMMs and coordination cannot be measured as single, unified constructs using one unobtrusive measure. Instead, different measures may provide information about specific aspects of these processes. Future research should begin with task analysis to identify relevant aspects of team SA and derive appropriate measures for the specific task environment. Task-related behavioural actions also represent a potentially valuable, yet often overlooked, category of unobtrusive measures. A key limitation is the substantial variation in how SMMs and coordination were operationalised and measured, making direct comparisons difficult. Future work should investigate how identified measures relate directly to team SA using validated measures.

## 1 Introduction

Professionals increasingly work in teams. An essential component of good teamwork is situation awareness (SA), especially in time-critical situations involving dynamically changing information (Endsley, 1999; e.g., aircraft maintenance teams: Endsley and Robertson, 2000). SA is often defined as the perception of a stimulus, its comprehension, and the projection of the future environmental state (the 3-Level model of SA; Endsley, 1995). In a team, not only should each teammate have adequate SA to perform their individual task, but also SA about the needs of their teammates—referred to as *team SA* (Salmon et al., 2008). A team is defined as a group of at least two individuals with a shared goal and specified roles working interdependently and adapting to a dynamic situation (Salas et al., 1992). Continuous measurement of team SA during teamwork may help reduce errors and improve safety. Yet, current measurement methods are not optimal for this purpose.

Currently, team SA is typically assessed using questionnaires or interviews gathered after task completion (e.g., Mansikka et al., 2021). A key limitation of these after-action reviews is that teammates may not accurately recall all details of their performance. An alternative method is to collect a probe during brief interruptions of the task (e.g., SAGAT, Situation Awareness Global Assessment Technique; Endsley, 2020). However, this approach may alter team SA itself (Salmon et al., 2006). To overcome these flaws, team SA should ideally be assessed in a more unobtrusive, objective, and continuous way—hereafter referred to as *unobtrusive measurement*. “Unobtrusive” refers to not interfering with or altering teaming processes by teammates answering questions during the task (Salmon et al., 2006). “Objective” means that the method should produce quantifiable data, as subjective judgements of SA (e.g., self-ratings or external assessors) may be influenced by cognitive bias (Salmon et al., 2006). Finally, “continuous” indicates that the measurement allows for the evaluation of team SA development throughout a task. To advance the unobtrusive measurement of team SA, the most likely option is the use of behavioral and physiological tools (Zhang et al., 2023).

### 1.1 Unobtrusive measurement of team SA through behavior and physiology

Technological advancements in wearable technology enable the continuous recording of physiological signals and behavioral data in a relatively unobtrusive manner (reviews: Kazi et al., 2021; Hałgas et al., 2023; Zhang et al., 2023). Such approaches are attractive for monitoring team SA as they do not interrupt the task at hand to do so. These methods have been investigated relating to individual SA. At the individual level, eye-tracking and electroencephalography (EEG) show promise—eye-gaze is a proxy of (visual) stimulus perception, and EEG may be indicative of stimulus comprehension (review: Zhang et al., 2023; application: Yang et al., 2024). However, team SA is more complex. It depends not only on each teammate's ability to develop their own SA, but also on teaming interactions. Consequently, unobtrusive measures of team SA need to capture these interactions (Guastello et al., 2020; Cooke et al., 2024). Below, we overview how behavioral data and physiological signals may relate to team SA.

#### 1.1.1 Behavioral data's role in measuring team SA

Human behavior is not uniform across situations—instead, it is influenced by the context in which the human is situated (Eiler et al., 2013). Thus, observable—and measurable—behavior can be indicative of changes in the way we interact with elements and with other participants in the environment. In this review, we will explore the use of verbal communication, eye-tracking, and bodily behavior as indicators of team SA. Verbal communication facilitates the sharing of information, potentially directly revealing the team's knowledge, and aids in coordinating team actions. For example, teammates that have good awareness of each other's SA-related needs may ask their teammates less questions (Demir et al., 2017). Next, as shown for individual SA, eye-tracking is an objective measure of (visual) attention allocation. For example, this can inform whether teammates are attentive to each other's needs. Last, bodily behavior may be influenced by other teammates' behavior. For example, it has been shown that when observing others, individuals may begin to mimic their actions (i.e., motor mimicry) or feel and express the same emotions (i.e., emotional contagion). This happens as a result of *empathy*—the process by which humans (and other animals) understand others' cognitive or emotional states when observing them (de Waal and Preston, 2017). Humans are especially attentive to others that are socially significant to them, such as family, friends, or teammates. Thus, similarity in teammate bodily behavior may be indicative of a close social bond and active social processing of teammate needs.

#### 1.1.2 Physiological signals' role in measuring team SA

Physiological signals stem from autonomous (ANS) and central nervous system (CNS) activity. While physiological processes are less directly related to higher-order constructs like team SA, they provide objective information about stress, mental workload, cognitive resource allocation, attentional engagement, and others. These factors are indirectly associated with (the capability to develop) team SA. In addition, similar to behavior, physiological activity occurs within a social environment. As such, it is affected by and reflective of changes in the environment (Waldman et al., 2019). The tools explored in this review are EEG, functional near-infrared spectroscopy (fNIRS), cardiac and breathing responses, and electrodermal activity (EDA). EEG and fNIRS reflect CNS activity, such as executive functioning and the engagement of socially relevant brain areas. These tools can inform about the extent to which teammates are processing information or actively interacting. Cardiac and breathing responses reflect sympathetic and parasympathetic ANS activity, while EDA primarily reflects sympathetic ANS activity. In general, *lower* sympathetic and *higher* parasympathetic ANS activity is associated with reduced stress and, consequently, greater availability of executive function resources and improved task performance. In contrast, *higher* sympathetic and *lower* parasympathetic ANS activity reflects elevated stress, which can hinder team processes. In some tasks, optimal performance has been shown to be associated with an *intermediate* level of sympathetic ANS activity (i.e., stress). Furthermore, the type of stress experienced (e.g., acute or chronic) and the nature of the task can also play an important role (Sandi, 2013). Alternatively, parallel stress responses can inform about similarity in teammates' mental processing, indicating a shared interpretation of the task environment. Overall, cardiac and fNIRS responses have lower temporal resolution, while EEG and EDA have higher temporal resolution, being able to follow rapid physiological changes (Boucsein, 2012). These characteristics are especially relevant in highly dynamic tasks.

#### 1.1.3 Interpersonal synchrony's role

As briefly mentioned, measuring team SA would require a method that may capture teaming interaction processes. While we do not specifically target interpersonal synchrony (IPS) in our review, we observed a strong presence of this construct in literature aimed at quantifying team processes. IPS refers to interdependent variation and temporal alignment in physiological or behavioral activity between at least two people (Palumbo et al., 2017; Mayo and Gordon, 2020; daSilva and Wood, 2025). IPS can be applied to various measurement modes, such as synchrony in ANS activity (e.g., heart rate, breathing rate, or EDA; Palumbo et al., 2017) or in brain activity (Montague et al., 2002). This can inform about various processes, such as common coordination styles or joint teammate attentiveness behavior. IPS has already been explored extensively in relation to teaming processes such as team coordination (Hałgas et al., 2023).

### 1.2 Goal of systematic review

The goal of this paper is to uncover behavioral and physiological measurement technologies of team SA. To achieve this, we systematically reviewed the literature for a range of behavioral and physiological indicators that may co-occur with strong team SA. The results of this review may advance unobtrusive measures of team SA and be used as a guide for future studies when selecting the most appropriate measures of the construct.

An initial search for literature on behavioral and physiological measurement of the construct “team SA” identified only one suitable study—linking team SA to electrodermal activity (EDA; Guastello et al., 2022). This apparent lack of literature likely reflects the complexity of team SA, which cannot be easily captured by a single unobtrusive measure and across all task contexts. To overcome this challenge, the perspective of this review shifted toward identifying behavioral and physiological measurements of *underlying team processes of team SA* that may be more readily measurable. The theoretical framework by Salmon et al. (2008) was used to identify these relevant processes. In this way, we uncovered potential measures that in future may be compared with traditional measurements of the broader construct of team SA (e.g., SAGAT).

### 1.3 Theoretical framework of team SA

Various conceptual frameworks of team SA have been proposed (see reviews: Salmon et al., 2008; Stanton et al., 2017). From these, we selected the framework described by Salmon et al. (2008) because of its relative simplicity and because we deemed the team processes involved to be potentially unobtrusively measurable. The framework has overlap with the team SA framework by Endsley and Jones (2001), which has been described as most well-known in the field (Salmon et al., 2008), but seemed too elaborate for our current purpose. In Salmon et al. (2008) framework, data from the environment are first processed by each individual teammate (based on Endsley's 3-Level model) and then transferred between teammates through team processes. These team processes, as well as the final product (team SA), may in turn influence the individual SA of each teammate.

The framework distinguishes four team processes: (1) shared mental models (SMMs), (2) collaboration, (3) co-operation, and (4) communication. An SMM is a common knowledge structure about the goals, expectations, and execution of a team task (Cannon-Bowers et al., 1993). Salmon et al. (2008) discuss collaboration, co-operation, and coordination as relevant processes. These concepts all have strong conceptual similarities (reviews: Bedwell et al., 2012; Castañer and Oliveira, 2020; dictionary definition: Merriam-Webster (n.d.)). They are encompassed by the definition—to work jointly with another party to achieve a common, valued goal. Salmon et al. (2008) do not distinguish or elaborate on why all three processes are uniquely relevant. Thus, we summarize them by the term *team coordination*—as it is the most used term in this field (e.g., Gorman et al., 2006; Demir et al., 2017). We note that SMMs and team coordination are theoretically intertwined: (1) strong shared knowledge enables the alignment in team expectations and information needs, resulting in better team coordination (Cannon-Bowers et al., 1993), and (2) better team coordination allows for better planning and transmission of information, therewith strengthening SMMs (Stout et al., 1999).

Last, communication represents transactive behaviors (verbal or non-verbal) by which teammates continuously and concurrently exchange information to advance shared meaning (Harris and Sherblom, 2018). We considered communication to be a behavioral measure of the other two processes. Therefore, our adapted framework focused on SMMs and team coordination (Figure 1).

**Figure 1:**
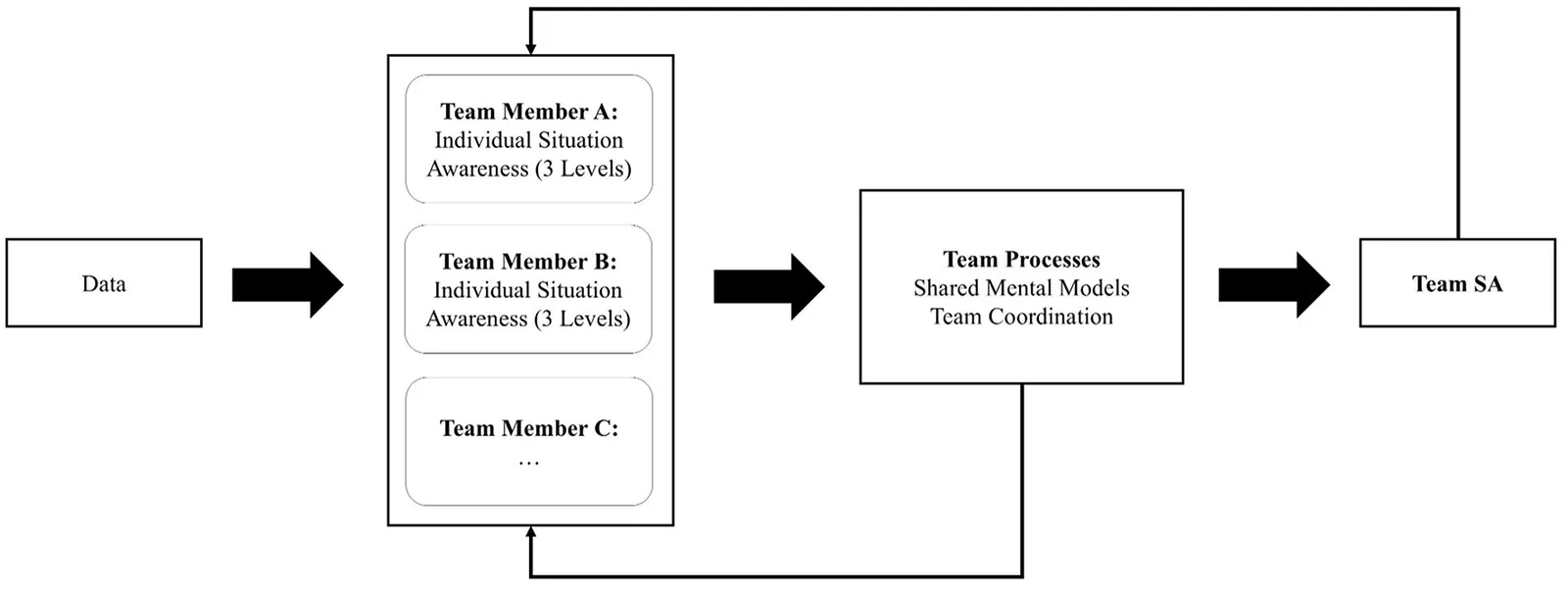
Framework for team situation awareness, adapted from Salmon et al. (2008).

## 2 Literature review

### 2.1 Methods

Based on our adapted model from Salmon et al. (2008; Figure 1), we searched the literature for unobtrusive measures reflecting the team processes *SMMs* and *team coordination*. We performed four separate scans of the scientific literature using different sets of keywords (see Table 1 for the keyword sets). Set 1 contained synonyms of SMMs and Set 2 contained synonyms of coordination (coordination synonyms derived from the reviews: Bedwell et al., 2012; Castañer and Oliveira, 2020). Set 3 contained behavioral and physiological measures (keywords selected after Zhang et al., 2023). We include verbal indicators even if they are currently marked by raters (i.e., not objective), due to their potential to be automatically transcribed (e.g., the Whisper tool, Radford et al., 2023) and analyzed (e.g., the Linguistic Inquiry and Word Count—LIWC-22—tool; Pennebaker et al., 2001). Set 4 contained keywords related to task-related behavioral actions as a measure of the team processes. Keywords inside a set were combined using an OR operator. Using Set 0, we filtered out non-human agents teaming. We searched Web of Science for peer-reviewed, English-language journal articles and conference proceedings published between 2000 and 2025.

**Table 1:** Keywords used in the systematic literature search of unobtrusive measures of the team processes in team SA.

Search set	Keywords
Set 1 SMMs	“team cognition,” “team mental model^*^,” “shared mental model^*^”
Set 2 Team coordination	*teamwork, collaboration, cooperation, coordination*
Set 3 Measures	physiolog^*^, “eye?track^*^,” respiration, “blood pressure,” electrocardioac^*^, “heart rate,” “heart rate variability,” electroencephalography, “brain signal,” “brain activity,” “event?related brain potential^*^,” “functional near?infrared spectroscopy,” electrodermal, “tissue blood volume,” “skin conductance level,” “skin conductance response^*^,” “galvanic skin response^*^,” “facial expression^*^,” speech, verbal
Set 4	“user interface,” UI, GUI, “system interface,” “control input^*^,” “user interaction^*^,” dashboard, “display interface,” “computer interface,” “digital interface”
Set 0	*robot, AI, synthetic, autonomous, human-machine*

An asterisk (^*^) denotes a wildcard, meaning that all words that begin with the indicated root are to be included in the results (example: physiolog^*^ indicates that physiology, physiological, physiologically will all be included). Quotes (“”) indicate that the exact written phase must be matched in the results (example: papers that contain mental model will not be included, but papers containing shared mental model will be). A question mark (?) indicates that any symbol may be included in its place (example: eye?track indicates that eye-track, eye track will all be included). UI, user interface; GUI, graphic user interface; AI, artificial intelligence.

The first two scans were performed on May 14th, 2025, titled Search 1 and Search 2. A simplified visualization of the two literature scans is shown in Figure 2. In Search 1 on measures of SMMs, we combined (with an AND operator) Set 1 (SMMs) with Set 3 (measures) and the keyword “team” (to limit records to studies on team dynamics) and excluded records with Set 0 keywords (NOT operator). In Search 2 on measures of team coordination, we combined Set 2 (team coordination) with Set 3 (measures) and with “team,” excluding records with Set 0 keywords. These first two scans revealed 47 records regarding team SMMs (Search 1) and 1,107 records regarding team coordination (Search 2). Upon observing a lack of task-related behavioral actions, such as key presses and display interactions, we ran two additional scans on July 17th, 2025, titled Search 1.2 and Search 2.2. Search 1.2 combined Set 1 (SMMs) and Set 4 (task-related actions) with “team,” excluding Set 0 keywords. Search 2.2 combined keywords from Set 2 (team coordination) with Set 4 (task-related behavioral actions) and “team,” excluding Set 0 keywords. In both additional scans, the keyword “human-machine” was removed from Set 0 to avoid interference with the added keywords in Set 4. We identified five records on team SMMs (Search 1.2) and 385 records on coordination (Search 2.2).

**Figure 2:**
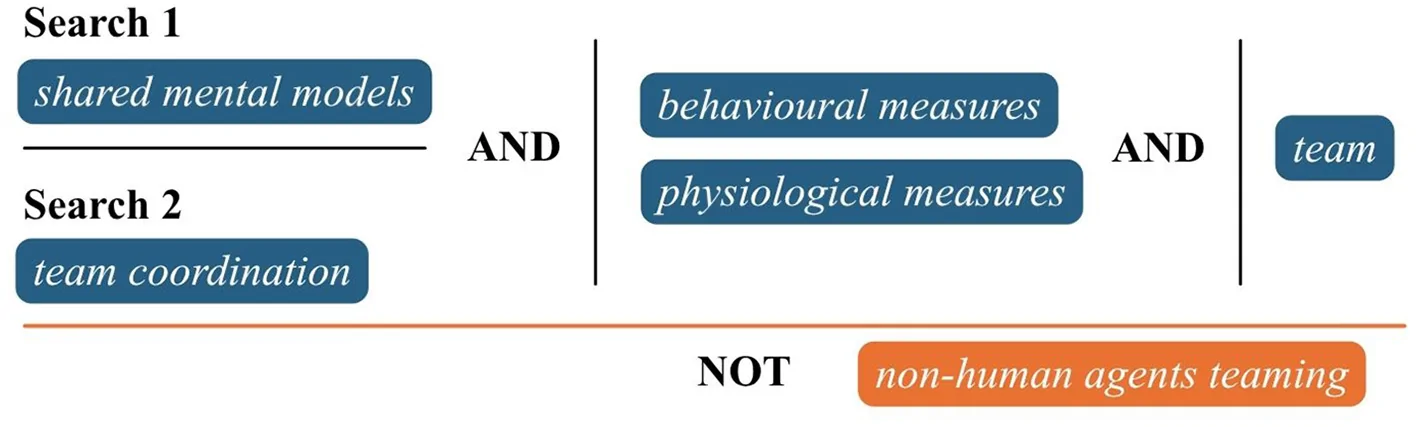
Visualization of the combination of keywords and operators in the performed scans.

### 2.2 Study selection

We followed the Preferred Reporting Items for Systematic Reviews and Meta-Analyses (PRISMA) statement (Moher et al., 2009), aimed at standardizing the reporting of systematic reviews. We utilized four selection phases: (1) identification of records, (2) initial screening (title and abstract), (3) eligibility assessment, and (4) included papers (flowchart shown in Figure 3). In the initial screening, we excluded all records that: (1) did not relate a measure from Sets 3 or 4 to a process from Sets 1 or 2; (2) were purely theoretical or conceptual; or (3) tested human and non-human agent teaming. All records were screened, and all information was extracted from the papers by one reviewer (EZ). To include a record in the full analysis, we adhered to the following inclusion criteria:

- SMMs or team coordination were discussed as the construct of interest, and not as an adjacent process (e.g., team performance, engagement, trust, etc.); and
- At least one unobtrusive measure was tested for a possible association with the targeted team process.

**Figure 3:**
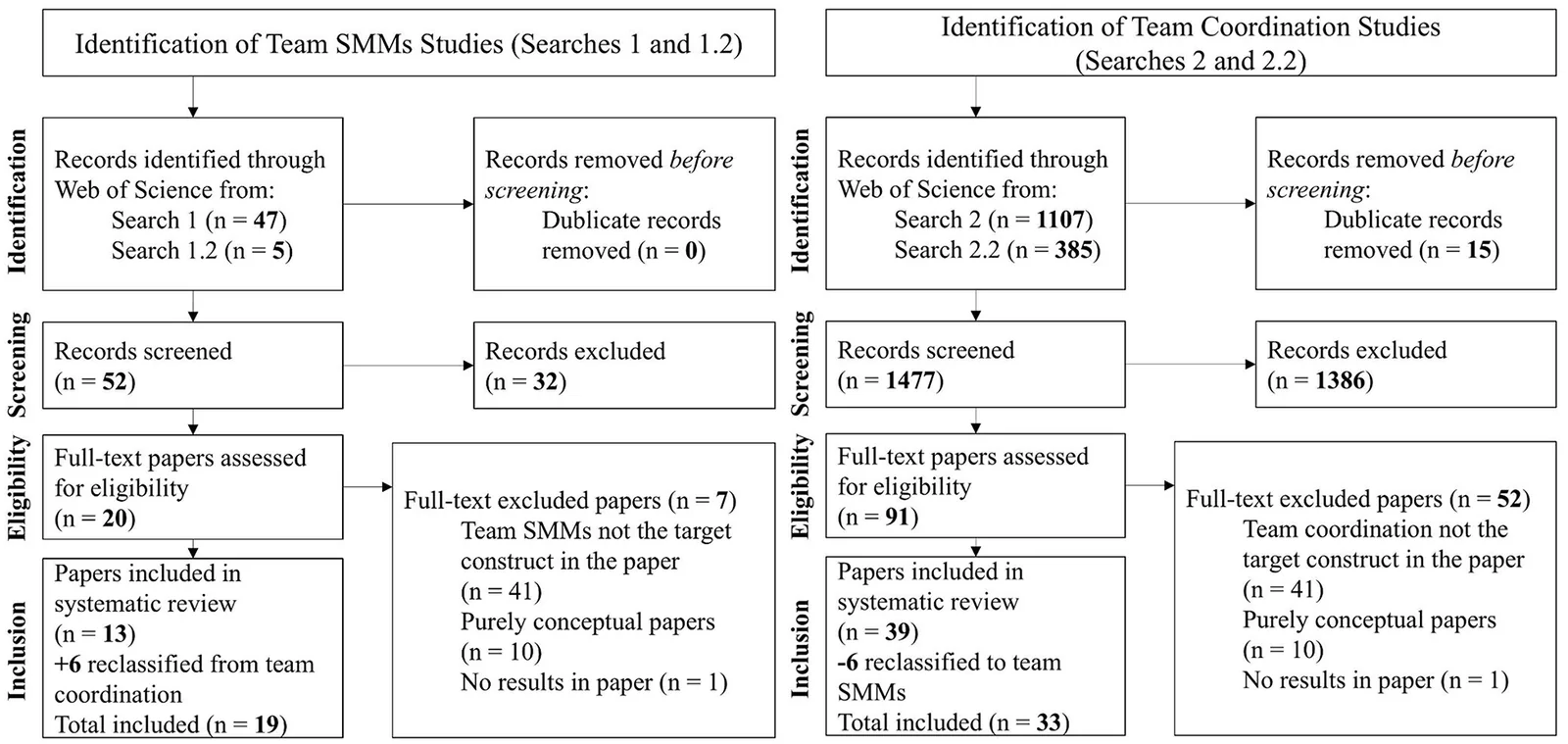
The flow of selection phases (suggested by PRISMA) from the identification of records to inclusion. Six studies initially identified through Searches 2 and 2.2 were reclassified to the results of Searches 1 and 1.2 after full-text review. These studies remain part of the final systematic review and are not counted as exclusions from the Searches 2 and 2.2 PRISMA diagram.

This approach led to the exclusion of records that: (1) only addressed correlates of general team performance or cohesion (e.g., Guastello et al., 2020) and: (2) the exclusion of the single paper that targeted team SA measures directly (Guastello et al., 2022). Following full-text assessment, we reallocated six records that were originally found under the team coordination search to SMMs (Amon et al., 2019; Vrzakova et al., 2019; Hoyle et al., 2020; Stewart et al., 2021; Pugh et al., 2022; del Rosario et al., 2024).

### 2.3 Extracted factors

For our review, we extracted the following information from each paper:

- **Sample and team size**: the paper's total sample size and the number of individuals in a team;
- **Task**: task environment;
- **Measure:** the main type of measure (e.g., “communication content”), where necessary, accompanied by how it was determined (e.g., “verbal content types were manually coded”). A relatively large number of papers (five for team SMMs and 23 for coordination) included measures of IPS;
- **Operationalisation of SMMs/coordination**: We recognized the following methods by which the authors estimated the strength of SMMs and team coordination: (1) subjective assessment by experts; (2) time on task, where it is assumed that SMMs and team coordination improve with task progression; (3) self-report questionnaire; (4) participant experience, where experienced teams (comprised of teammates who are experts on the task, or of teammates who have worked together before) are assumed to have stronger SMMs and coordination than inexperienced teams; (5) experimentally controlled methods, for example by manipulating the amount of information shared within a team; (6) methods associated with a task phase, where it is assumed that certain task phases require strong SMMs; and (7) performance-associated methods, where it is assumed that better task performance indicates a stronger SMMs. For team coordination, there was an additional category: (8) teaming conditions (task conditions requiring active teamwork, compared to individual work or other baseline conditions); and
- **Result:** relationships between the unobtrusive measure and SMMs/team coordination.

Results are discussed and grouped according to measuring modes, starting with behavioral measures: (1) verbal communication; (2) eye-tracking; (3) bodily behavior (facial expressions, head movements). Then, the results of all physiological measures will be described: (4) cardiac measures; (5) EEG; (6) functional near-infrared spectroscopy (fNIRS); (7) breathing measures; (8) EDA. Finally, (9) we describe the papers that combined multimodal measurement into a unified measure (all other papers are discussed in each corresponding mode). All findings on individual physiological and behavioral activity, as well as IPS findings, are discussed within their respective measurement modes, while overarching IPS findings that link the different modes are presented separately in section (10) IPS findings across measuring modes. These global trends in IPS can reveal general patterns of convergence across team processes.

A brief overview of the papers, categorized by team process(es) (SMMs and/or team coordination), measurement mode(s) (behavior, physiology, and/or multiple modes of measurement), and type(s) of measurement(s) (e.g., verbal communication) can be found in Supplementary Table 1.

## 3 Results

### 3.1 Study characteristics

After applying the inclusion criteria and paper reallocation, 19 studies remained on team SMMs and 33 on team coordination. There was a large variation in the investigated tasks, including laboratory cognitive tasks (gaming, programming, creative design, moral dilemma); controlled physical tasks (piano playing, juggling); and research conducted in real-life environments with large freedom in behavior (surgery, basketball, firefighter training). We observed both virtual (e.g., CS:GO players in virtual teams; Eldadi and Tenenbaum, 2025) and non-virtual teaming (e.g., teams in one room solving a product design problem; Badke-Schaub et al., 2011) in all measurement modes—meaning that the types of measures chosen were not specific to virtual or non-virtual teaming (Vorreuther et al., 2026).

The most common methods to estimate the strength of SMMs were: participant experience (used in 26% of the SMM studies); association with task phase (21%); and subjective assessment by experts (21%). For coordination, these were: teaming conditions (48% of the coordination studies), and subjective assessment by experts (30%). An overview of all methods used to estimate SMMs and team coordination is shown in Figure 4.

**Figure 4:**
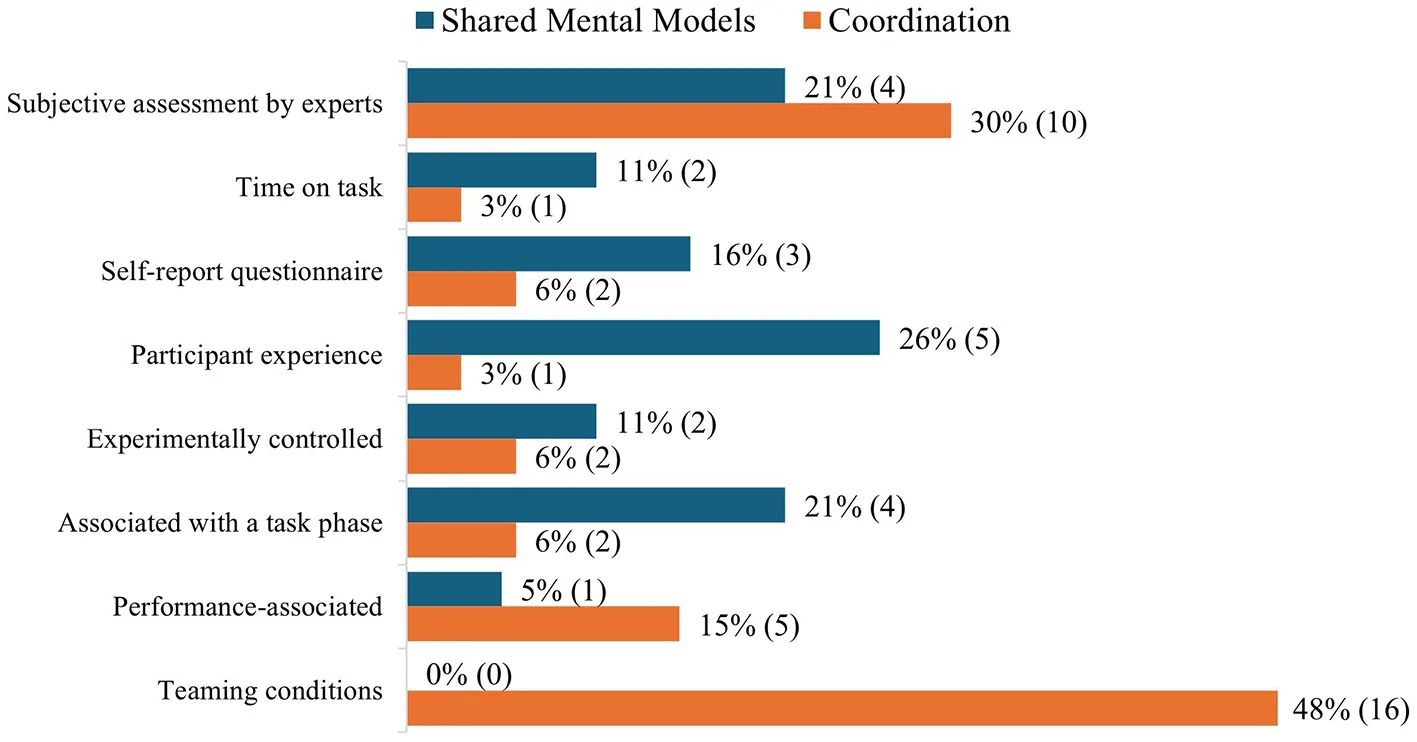
Percentages (out of the total number of studies per team process) and number of studies (in brackets) of the categories of team process operationalisation. Some studies employed multiple operationalisations of a team process.

Most studies on SMMs investigated verbal communication measures (47% of the SMM studies) and cardiac measures (42%). For team coordination, the most popular modes were cardiac measures (30% of the coordination studies) and fNIRS (27%). We did not find studies that specifically mentioned task-related behavioral actions as indicators of team processes. The frequency of the measuring modes described is shown in Figure 5.

**Figure 5:**
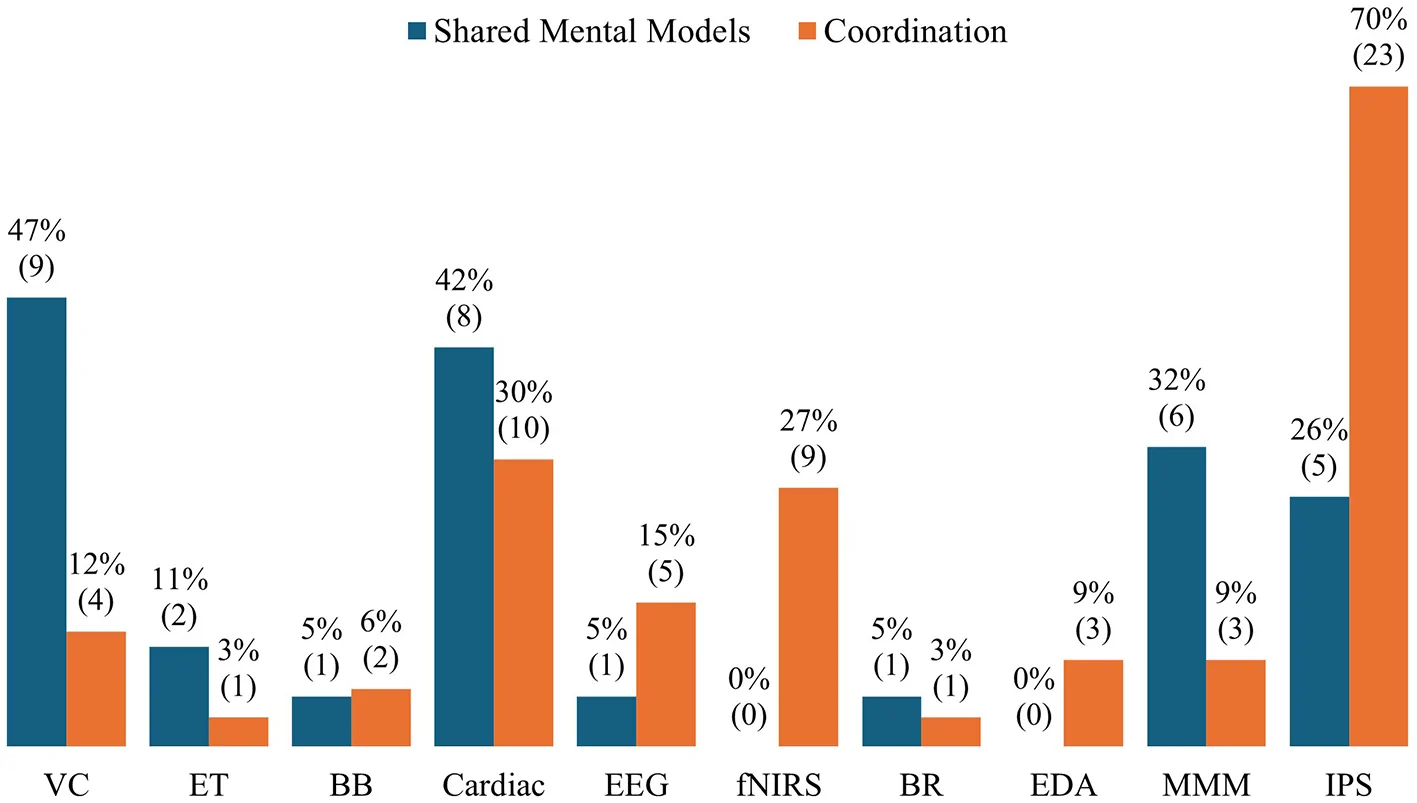
Percentages (out of the total number of studies per team process) and number of studies (in brackets) of the measuring modes (and IPS) used per team process. Studies using multiple modes are represented in all respective modes and in MMM. Studies using PS are represented both in IPS and in the mode(s) for which IPS was calculated. VC, verbal communication; ET, eye-tracking; BB, bodily behavior; EEG, electroencephalography; fNIRS, functional near-infrared spectroscopy; BR, breathing; EDA, electrodermal activity; MMM, multiple modes of measurement; IPS, interpersonal synchrony.

Ten studies explicitly tested whether team SMMs or team coordination were related to team performance. Most of these relations were positive (two studies for SMMs: Espevik et al., 2011; Bönsel and Müller, 2021; three for coordination: Xue et al., 2018; Mayseless et al., 2019; Sun et al., 2021). Some studies reported negative associations (one for SMMs: Hoyle et al., 2020, Study 3; and for coordination: Fusaroli et al., 2016), or non-significant (SMMs, though the trend was positive: Espevik et al., 2006; two studies on coordination: Henning et al., 2001; Hoyle et al., 2020, Study 1). The rest of the included studies did not explicitly test the relation between team processes and team performance.

In the subsequent sections, we synthesize the promising findings concerning measures of SMMs and, respectively, team coordination per measuring mode (e.g., eye-tracking, fNIRS). In the text, we also summarize non-significant results. All statistically significant measures are outlined in Supplementary Tables 2 (SMMs) and 3 (team coordination), grouped by measuring mode, summarizing information about sample size and the size of a team, the task, the operationalisation of the team process, the unobtrusive measure, and the observed association of the measure with the team process. All resulting associations are related to strong SMMs or coordination, unless stated otherwise.

### 3.2 Behavioral measures of SMMs

We now consider the literature on how measurements of behavior have been used to elucidate SMMs between team members, which is the first main component of the adapted model of team SA (Salmon et al., 2008).

#### 3.2.1 Verbal communication and SMMs

Verbal measures used to estimate team SMMs can be broadly divided into communication *content* (content of the communicated information), communication *dynamics* (e.g., transitions from one content category to another), and communication *frequency* (e.g., overall frequency of verbal utterances). We overview the findings in this order, starting with communication content and ending with communication frequency.

Although the included studies used slightly different coding schemes and terminology, many of the verbal communication categories overlapped conceptually. Therefore, to facilitate comparison across studies, we grouped similar categories under a common set of labels based on the terminology used in the literature:

- *task-relevant statements* (clarifications, task questions, explanations);
- *knowledge exchange* (e.g., factual task statements);
- *planning* (procedures, strategising, predictions, performance monitoring, like announcing intended actions);
- *action-oriented* (e.g., commands);
- *reflective* (evaluations);
- *motivational/emotional*;
- *uncertainty* (expressing doubt);
- *acknowledgment* (confirmatory—“Yeah,” or appreciative statements—“Good idea”); and
- any other statements, referred to as “*task-irrelevant*” by the studies (e.g., social or other statements without a clear task function).

According to three studies involving cognitive tasks (e.g., gaming, naval simulation), teams with strong SMMs (according to the authors' operationalisation of SMMs) exhibited *more* knowledge exchange statements (Espevik et al., 2011; Bönsel and Müller, 2021; Eldadi and Tenenbaum, 2025), acknowledgment statements (Eldadi and Tenenbaum, 2025), as well as more action-oriented, motivational/emotional, and uncertainty statements (Eldadi and Tenenbaum, 2025). Espevik et al. (2011) observed increased knowledge exchange only in physically distributed teams (i.e., where teammates worked in separate physical locations), but not in co-located teams. At the same time, teams with stronger SMM teams produced *fewer* task-irrelevant statements in two studies (Espevik et al., 2011; Eldadi and Tenenbaum, 2025); in Espevik et al. (2011), this effect was only observed in co-located, but not in physically distributed teams. Teams with stronger SMMs also used *fewer* planning statements (Badke-Schaub et al., 2011). A study in real-life football found that teams with self-reported strong SMMs exchanged fewer task-relevant statements (Blaser and Seiler, 2019).

On communication dynamics, we found:

- *communication patterns* (how teams transitioned from one content category to another);
- *content overlap* (congruency in the communication content between teammates); and
- what we refer to as *communication strategy* (unsolicited, compared to requested information).

We found two studies addressing communication patterns: one in online gaming (Eldadi and Tenenbaum, 2025), and another in tennis players (Lausic et al., 2009). These studies suggest that transitions between content categories can provide insight into how teammates coordinate and respond to each other's contributions. For instance, action-oriented statements were followed by other action-oriented statements in teams with strong SMMs (operationalised through more experienced teams in Eldadi and Tenenbaum, 2025; and through higher performing teams in Lausic et al., 2009). Another study (Mamykina et al., 2016) investigated communication overlap in clinical teams, finding that during task phases requiring strong SMMs—such as patient handovers—teams exhibited a high degree of overlap in the communicated content.

Regarding communication strategy, Espevik et al. (2011) found that teams with strong SMMs (i.e., more experienced teams) pushed more information (offered unsolicited information), compared to requested information (explicitly asked teammates for information). Espevik et al. (2006) also found a decrease in requested information in strong (i.e., more experienced) SMM teams.

Finally, regarding verbal communication frequency, studies used:

- *overall verbal frequency* (total speech units, regardless of verbal content);
- *pace* (word count per second); and
- distribution of responsibility between members for sharing and discussing information.

We found varying results on overall verbal frequency. Three studies reported a decrease in verbal frequency in teams with strong SMMs (Espevik et al., 2006; Blaser and Seiler, 2019; Bönsel and Müller, 2021). Another study on online gaming found that verbal frequency increased with strong SMMs (Eldadi and Tenenbaum, 2025). In the naval simulation study by Espevik et al. (2011), distributed teams with stronger SMMs demonstrated a higher frequency of task-relevant statements compared to co-located teams. This effect was even more pronounced under challenging conditions. According to the authors, teams with stronger SMMs were better able to adapt to the distributed teaming environment. Weaker SMMs teams maintained the same amount of verbal communication despite their teaming environment.

Eldadi and Tenenbaum (2025) also found that strong-SMM teams communicated at a faster pace. Bönsel and Müller (2021) related (self-reported) strong-SMM teams to equal communication distribution, suggesting knowledge similarity, which was not apparent in the study of Eldadi and Tenenbaum (2025).

Pugh et al. (2022) investigated the accuracy of a language-based model in distinguishing SMM-related verbal content from other verbal content. The model successfully distinguished instances of shared knowledge communication from other types of communication (coordinating and maintaining team function).

Overall, teams with stronger SMMs appear to exhibit more structured communication, characterized by regular communication patterns. They also tend to be more action-oriented and affirm the contributions of their teammates. We found indications of high communication content overlap and a faster speaking pace in strong-SMM teams. Stronger SMMs were associated with a communication strategy (more pushing, compared to requesting information). Some of the verbal measures used in the studies, such as communication content, overall verbal frequency, and distribution, showed inconclusive associations with SMMs. We overview potential reasons for differences between the studies in the discussion section.

#### 3.2.2 Eye-tracking and SMMs

Two studies employed eye-tracking in relation to SMMs. Espevik et al. (2011) found that teams with stronger SMMs, compared to weaker, more frequently directed their gaze toward areas of the digital display associated with their teammate's actions or other points of interest. This is an indicator of performance monitoring. In a study in visual programming, Vrzakova et al. (2019) found that teams exhibited stronger SMMs (as rated by experts) when teammates consistently directed their gaze toward changes on the display (e.g., the appearance of a new stimulus). However, IPS-eye-gaze between teammates in response to display changes was not significantly associated with SMMs.

#### 3.2.3 Bodily behavior and SMMs

We found only one paper relating bodily behavior to SMMs. In a basketball game, Bourbousson et al. (2015) constructed a team social network by interviewing teammates and analyzing video recordings of the bodily behavior of teammates. In this way, they determined whether individuals directed their attention toward partner teammates while planning their actions. This analysis encompassed elements such as body direction, head movements, and gaze direction, as well as teammates' reported interpretation of their actions at the time. In teams with stronger SMMs (operationalised through participant experience), teammates did not explicitly direct their attention toward other teammates while planning their actions. This finding was reflected by low network density (i.e., how many connections out of the total possible existed in the network), low network outdegree (i.e., how many connections a node has), and low network reciprocity (i.e., the extent to which connections are mutual). The authors concluded that teammates relied on SMMs and anticipation of future developments, thereby reducing the need for continuous, overt monitoring of one another.

### 3.3 Physiological measures of SMMs

We now follow with physiological measures and their relationship to team SMMs.

#### 3.3.1 Cardiac measures and SMMs

In several studies on SMMs, authors examined both individual and IPS trends in HR (beats per minute) and HRV (fluctuations in HR quantified using measures such as the root mean square of successive differences between inter-beat intervals, RMSSD). Higher stress or arousal (i.e., sympathetic ANS activity) is related to increased HR and decreased HRV, while calmness (i.e., parasympathetic ANS activity) is associated with decreased HR and increased HRV. Two studies found relatively stable HR in teams with strong SMM throughout a naval simulation task despite increasing task challenge (Espevik et al., 2006, 2011). HR also quickly recovered after challenging conditions (Espevik et al., 2011). In accordance with this, teams with stronger SMMs showed high HRV (SMMs were operationalised through self-reports in Sætrevik, 2015: Study 2; and through time on task in Hoyle et al., 2020, Study 3). According to the authors, this reflected greater availability of cognitive resources.

Regarding IPS-HR, one paper found positive correlations between teammates' HR in a psychomotor task (juggling; Filho et al., 2017). Similarly, del Rosario et al. (2024) reported IPS-HR between teammates during real-time surgery. Although del Rosario et al. (2024) did not explicitly examine SMMs, they examined shared motivation, emotions, and stress, where shared motivation falls under SMMs (Cannon-Bowers et al., 1993). IPS indicators were also found for HRV. Kennedy-Metz et al. (2021a) found IPS-HRV, namely, synchronized negative RMSSD peaks between teammates during challenging real-time surgery phases. They argued that this reflected SMMs and coordinated action. Finally, one paper found that teammates had a statistically similar average HRV during a challenging surgical phase that required strong SMMs (Kennedy-Metz et al., 2021b). The authors interpreted this to support a similar psychological state between teammates during the formation of SMMs.

Overall, the studies associate cardiac measures with SMMs, suggesting that reduced stress, greater availability of executive function resources, and faster recovery following challenging task phases are linked to stronger SMMs. IPS-HR and IPS-HRV indicated shared mental states.

#### 3.3.2 Electroencephalography and SMMs

Using EEG, Hoyle et al. (2020; Study 3) found increased absolute theta power in the frontal regions with prolonged engagement in an online football game. Absolute theta power represents the spectral power density in the theta 4–8 Hz power band per EEG channel or a cluster of channels—in this case, in the frontal channels. The authors reasoned that this reflected heightened attention, workload, and stronger SMMs.

#### 3.3.3 Breathing measures and SMMs

No studies examined the relationship between individual breathing activity and SMMs; however, one study related IPS in breathing rate (IPS-BR) between teammates to SMMs. Breathing rate refers to the number of breaths taken per minute. IPS-BR positively correlated with SMMs among juggling teammates, but only in the easy condition (Filho et al., 2017). The authors interpreted these findings as indicating that the challenging condition significantly constrained the team's ability to establish sufficiently strong SMMs, thereby leading to decreased IPS-BR.

### 3.4 Multimodal integration of SMMs measures

Six studies assessed SMMs across multiple modes (Espevik et al., 2006, 2011; Filho et al., 2017; Amon et al., 2019; Hoyle et al., 2020: Study 3; Stewart et al., 2021), two of which further integrated these modes into a single composite measure (Amon et al., 2019; Stewart et al., 2021). As this approach extends beyond multimodal measurement alone, this section evaluates the results of the unified measures.

Amon et al. (2019) integrated data on body movements, speech rate, and display changes to create a single measure of *behavioral regularity* during a computer programming task. Behavioral *irregularity* was negatively related to SMMs, as rated by an observer. Stewart et al. (2021) employed multimodal classification models incorporating linguistic, acoustic-prosodic, facial expression, and task context features to identify different aspects of collaborative problem-solving. Based on these features, their model could distinguish ‘*construction of shared knowledge*' from ‘*negotiation/coordination*,' and ‘*maintaining team functions*.'

Overall, a few multimodal approaches successfully integrated complementary measures, which showed promise in measuring the complexity of SMMs.

### 3.5 Interpersonal synchrony findings across measurement modes for SMMs

Out of the 19 total studies, three examined how ANS activity co-varied between teammates (Filho et al., 2017; Kennedy-Metz et al., 2021a; del Rosario et al., 2024), while two investigated IPS in teammates' behavior (Amon et al., 2019; Vrzakova et al., 2019). There were different approaches to calculating IPS (see Supplementary Table 2 for details per paper), each with their own advantages and disadvantages—which is beyond the scope of the current review. Across approaches, IPS was positively associated with team SMMs. In two studies, IPS was a particularly good indicator of SMMs during challenging conditions (Kennedy-Metz et al., 2021a; del Rosario et al., 2024), while others observed high IPS throughout tasks (Filho et al., 2017; Amon et al., 2019; Vrzakova et al., 2019).

### 3.6 Behavioral measures of team coordination

Now, we consider the literature on how measurements of behavior have been used to elucidate team coordination, being the second important component of our adapted model of team SA.

#### 3.6.1 Verbal communication and team coordination

We found the following verbal measures to estimate team coordination:

- *speaking* (detected voice activity) *before action*;
- *prosodic accommodation* (adjusting, e.g., pitch range and speech rate, such as to match their teammate's);
- *focused listening* (one teammate speaking at a time);
- *simultaneous speech* (teammates speaking at the same time),
- and *total speaking* (spoken communication units) or *silence time* (silence units).

Teams with strong coordination (as assessed through each paper's operationalisation method) showed several communication behaviors. They spoke before acting in firefighter training (Feese et al., 2013) and showed prosodic accommodation in a crew resource management task (De Looze et al., 2015). Additionally, they showed more focused listening in an online football simulation game (Hoyle et al., 2020; Study 1).

Simultaneous speech, total speaking time, and total silence time showed conflicting results. Strongly coordinated teams had both *more* simultaneous speech (De Looze et al., 2015) and *less* simultaneous speech in an online football simulation game (Hoyle et al., 2020; Study 1). Hoyle et al. (2020; Study 1) further found a steady decrease in silence time with stronger coordination, while De Looze et al. (2015) found no significant link between speaking or silence time and coordination (assessed through experts). In Hoyle et al. (2020; Study 1), the relationship between total speaking time and team coordination was likewise inconclusive.

As with SMMs, Pugh et al. (2022) demonstrated high language-based model accuracy in distinguishing coordinating communication from other types of communication (e.g., sharing knowledge), indicating that verbal communication can be a useful measure of team coordination.

In summary, promising measures of team coordination include prosodic accommodation, focused listening, and speaking before action. In contrast, measures such as total speaking time, total silence time, and simultaneous speech appear to be more sensitive to contextual factors or to the specific operationalisation of team coordination.

#### 3.6.2 Eye-tracking and team coordination

Vrzakova et al. (2019) used eye-tracking to estimate team coordination (assessed through self-report and by experts). The authors presented individual activity in eye-gaze and display changes, as well as patterns in IPS-eye-gaze (see Supplementary Table 3 for a full overview). These measures reflect whether teammates direct their gaze toward relevant display changes; and whether they do so in a synchronized way. Overall, strong IPS in eye-gaze to display-changes behavior, as well as high regularity in eye-gaze-to-display-changes behavior, were positively related to team coordination. However, high complexity in eye-gaze (high fixation entropy) and display change patterns were negatively related to team coordination. These findings suggest that team coordination benefits from more synchronized and regular eye-gaze-to-display-changes behavior, while more complex and variable behavioral patterns are associated with poorer team coordination.

#### 3.6.3 Bodily behavior and team coordination

Two studies examined IPS in bodily behavior. In the first study, Strang et al. (2014) observed higher IPS in head-postural sway movements in real teams compared with an artificial team composed of individuals performing the puzzle task alone. No differences were found between real and false-matched teams (i.e., a member of one real team artificially matched with a member from another team). The authors interpreted this as reflecting a common coordination style across all teams, potentially driven by task constraints. In the second study, Mønster et al. (2016) examined IPS in facial muscle activity using electromyography, focusing on facial muscles related to smiling (zygomaticus activity) and frowning (corrugator supercilii activity). IPS-zygomaticus (smiling) was higher in real, compared to false-matched assembly-line task teams. No differences were observed for IPS-corrugator-supercilii (frowning). The authors stated that smiling may be more contagious than frowning. IPS-zygomaticus (smiling) correlated with positive, self-reported emotional coordination (i.e., a good relationship with teammates), but showed no relationship with performance-related coordination aspects.

### 3.7 Physiological measures of team coordination

We now elaborate on measures stemming from physiology and their connection to team coordination.

#### 3.7.1 Cardiac measures and team coordination

As for SMMs, team coordination was related to individual and IPS trends in HR and HRV.

During real-time surgery phases, demanding strong team coordination, teammates showed statistically similar averaged HRV (Kennedy-Metz et al., 2021b). In addition, high-coordination phases aligned with synchronized negative peaks in HRV between teammates (Kennedy-Metz et al., 2021a). Increased IPS-HRV in teams with strong coordination was also reported in two additional studies, conducted in a programming task and in real-time surgery, respectively (Veerabhadrappa et al., 2021; Powezka et al., 2022). During medical training sessions, higher IPS-HRV (calculated via the standard deviation of normal-to-normal intervals, SDNN) was observed in teams with stronger, compared to weaker coordination (operationalised through teaming conditions and time on task; Wespi et al., 2025). Among the two, subsequent high-coordination conditions, IPS-HRV was higher in the second task than in the first, suggesting a potential timing effect of IPS-HRV. Wespi et al. (2025) also reported lower IPS-HRV (calculated via RMSSD) in high- compared to low-coordination tasks. They interpreted this as reflecting higher stress and lower cognitive resources in the high-coordination tasks. Further, the authors noted that the RMSSD metric tends to be more variable under stress, which can reduce HRV-based IPS.

Regarding IPS-HR, Wespi et al. (2025) found increased IPS-HR in teams with strong coordination, interpreted as reflecting shared goals, effort, and strong coordination. In the same study, teams in closer physical proximity showed consistently higher HR-IPS. Timing effects in IPS-HR were also found within the high-coordination tasks, with lower IPS in the second high-coordination task, compared to the first—the opposite pattern of what was observed in IPS-HRV (HRV calculated through SDNN). Filho et al. (2017) found high HR IPS during expert-team juggling.

Strang et al. (2014) reported higher IPS-HR in real teams compared to false-matched teams in a puzzle task. IPS-HR was elevated in real teams relative to false-matched teams only when teammates shared the same role, whereas no such increase was observed when teammates held different roles. The authors interpreted this result as indicating that IPS-HR reflected similar intra-role coordination processes, but not general teaming processes.

Fusaroli et al. (2016, Study 1) found that IPS-HR was higher in false-matched teams than in real teams, and higher during individual construction task phases than during collective (team) task phases. However, IPS-HR was more stable in real teams and increased over time only in real teams engaged in collective tasks. The authors suggested that both task constraints (e.g., individual engagement) and coordinative effects contributed to this. Furthermore, Study 2 showed that IPS-HR was positively associated with behavioral and speech coordination (Fusaroli et al., 2016).

Two studies found no significant link between IPS-HR and team coordination, and the authors of both suggested that their experimental designs influenced this (Henning et al., 2001; Mønster et al., 2016). Henning et al. (2001) discussed their potential imprecise operationalisation of team coordination. For this reason, Henning et al. (2001)'s results are mentioned in the remaining relevant sections but not considered in the qualitative summaries of this review. Mønster et al. (2016) reasoned that their assembly-line task lacked sufficient arousal to elicit high IPS-HR.

Overall, findings on cardiac measures appear to be context-dependent and to have timing effects. Two studies indicated that strongly coordinated teams maintain synchronized levels of calmness, reflected in parasympathetic activity (measured through IPS-HRV). While IPS-HR may provide meaningful insights into coordination during challenging tasks, it also appears to be influenced by task-driven factors and not solely by coordinative processes.

#### 3.7.2 Electroencephalography and team coordination

All five EEG studies on team coordination examined IPS. IPS was associated with brain activity in regions related to social processing (through anterior cingulate cortex activity), attentional coordination and problem-solving (frontal areas), and sensory integration (parietal areas). Synchrony was observed in alpha (8–12 Hz, relaxation), beta (12–30 Hz, alertness and focus), and/or gamma (30–40 Hz; complex problem-solving) bands.

Dodel et al. (2011) and Reinero et al. (2021) operationalised team coordination through teaming conditions. Dodel et al. (2011) found that high IPS in the prefrontal cortex (PFC) was positively linked to team coordination in simulated combat. Reinero et al. (2021) reported a smaller IPS decline over time in frontal, occipital, and parietal areas (in the alpha and beta bands) for problem-solving teams with strong coordination, compared to weak.

Some studies reported asymmetrical IPS when one teammate assumed a leadership role, and the other followed. Astolfi et al. (2010) found IPS in the leader's dorsolateral prefrontal activity with the follower's anterior cingulate cortex and parietal activity in a collaborative card game task. No IPS occurred between non-teammates. The authors suggested that this pattern reflected that followers inferred the leaders' strategies, while leaders were actively formulating those strategies. In a virtual room clearing task, Dodel et al. (2020) reported IPS in low leader beta power with high follower beta power; and in low leader gamma power with low follower beta power during task phases with strong, compared to weak team coordination (team-performance-associated). Both effects were observed in medial-centro parietal locations. The authors state this reflected the unidirectional information flow from leader to follower, indicating high team coordination.

Finally, Zamm et al. (2021) linked temporal coordination in duet piano players to high IPS in oscillatory amplitude fluctuations at the performance frequency. This shows that IPS may be indicative of coordination dynamics.

Overall, despite varied study procedures, IPS in frontal (e.g., executive functioning), socially relevant regions, and in sensory integration regions (parietal) emerged as a key measure of team coordination. In decentralized teams—where no leadership hierarchy exists—coordination may be supported by symmetrical IPS, as members collaboratively propose and endorse plans of action. In contrast, in centralized teams, it is expected that complementary, rather than identical, brain regions synchronize between individuals. This shows the direction of information from leader to follower.

#### 3.7.3 Functional near-infrared spectroscopy and team coordination

fNIRS formed the largest category of team coordination measures. In the brain, fNIRS measures levels of oxygenated (HbO) and deoxygenated (HbR) hemoglobin, with higher HbO indicating greater neural activity in a specific region. Across studies, activity involved frontal areas linked to executive control, goal maintenance, coordination and the mirror neuron system (specifically—in the frontopolar and PFC; dorsolateral PFC; inferior frontal gyrus), motor areas (primary motor cortex), and social areas (specifically—temporo-parietal junction, TPJ, for perspective taking and joint attention; superior temporal gyrus, related to the mirror neuron system). The mirror neuron system (MNS) involves neurons that track and interpret the actions of others.

Hayne et al. (2023) and Liu et al. (2021) operationalised team coordination through teaming conditions. Hayne et al. (2023) found that in a risk-taking task, strong-coordination teams exhibited reduced activity in primary motor cortex (related to motor functions) and bilateral dorsolateral PFC. These regions are associated with goal maintenance, coordination, executive control, and the interpretation of others' actions. Left TPJ activity did not strictly relate to stronger team coordination. Yet, it was active when individuals were inferring others' goals and actions in the context of the task. Machine learning identified the left TPJ and primary motor cortex as the most discriminative regions for coordinative processes. Liu et al. (2021) used network and graph theory analyses in a group drumming task. Strong-coordination teams showed higher medial PFC Global and Local Network Efficiency, and higher left TPJ Global (but not Local) Network Efficiency. This indicated that strongly coordinated teams shared information broadly rather than only with neighboring teammates.

Two studies examined individual brain activity and synchronized activity between teammates (Xu et al., 2019; Zhang et al., 2021). Xu et al. (2019) tracked frontal activity (anterior PFC), related to the functions of executive control, goal maintenance, and interpreting others' actions. Strong coordination (operationalised through teaming conditions) was associated with increased activity related to these functions. This was expressed through slightly increased HbO in the regions toward the end of the joint drawing task requiring higher coordination. Three IPS-fNIRS findings also emerged in teams with strong coordination: increased IPS-fNIRS in the anterior PFC (expressed through high IPS-HbO); high IPS-fNIRS in decreased anterior PFC activity (IPS-HbR, although this effect was evident not only in stronger coordination, but in general in all task periods of shared information processing, even without active team coordination); and finally, high IPS-HbO/HbR in anterior PFC, increasing with challenging conditions, interpreted as signaling greater coordination demands. In a moral dilemma task, Zhang et al. (2021) found that individuals showed high dorsolateral PFC and middle frontopolar activity, suggesting increased inhibition of selfish behavior and increased coordination effort. Additionally, teams exhibited high IPS in the right inferior frontal gyrus, which the authors interpreted as reflecting efforts to understand teammates' intentions and align on shared goals.

Most studies relied on IPS-only measures. In strong-coordination teams using a moral dilemma task, Zhang et al. (2020) observed elevated IPS-fNIRS in the inferior frontal gyrus. This region is implicated in executive functioning, goal maintenance, and the interpretation of others' actions. The authors also found IPS-fNIRS in the dorsolateral PFC, related to similar functions, but this finding was not associated with strong team coordination. In Xue et al. (2018), expert-rated strong-coordination teams showed elevated IPS-fNIRS in the right dorsolateral PFC (also found by Li et al., 2020) and right TPJ—attributed to inferring teammate goals and actions during realistic creativity problem-solving. (Sun et al. 2021) studied joint drawing and found IPS-fNIRS in the left frontopolar regions, which implied synchronized executive functioning, goal maintenance, and interpretations of others' actions in performance-associated strong-coordination teams (a finding not significant in Li et al., 2020's joint drawing task). Liu et al. (2021) observed high IPS-fNIRS in the medial PFC in teams with stronger coordination (operationalised through teaming conditions), compared to weaker. They also observed the same (but non-significant) trend in IPS-fNIRS in the left TPJ during real-time drumming.

Mayseless et al. (2019) found that strong coordination in creative design was associated with asymmetrical IPS-fNIRS, meaning that linked activity occurred in different, rather than identical, brain regions between teammates. In this study, the asymmetrical IPS-fNIRS showed that teams synchronized between regions of executive control and the MNS/mentalizing regions. Strong-coordination teams showed high IPS-fNIRS between the dorsolateral PFC in one member and the posterior superior temporal gyrus in another member; IPS-fNIRS between the inferior frontal gyrus and the posterior superior temporal gyrus; and IPS-fNIRS between the anterior PFC and TPJ with marginal significance (*p* < 0.1). Anterior IPS-fNIRS in PFC to posterior superior temporal gyrus increased with stronger self-reported coordination, interpreted by the authors as reflecting verbal communication and coordination processes. Over time, IPS-fNIRS between the anterior PFC and TPJ decreased as externally rated coordination improved, suggesting less effort was needed to coordinate and that teammates increasingly anticipated each other's actions as they adapted.

While the dissimilar methods make summarizing results challenging, some findings can be generalized. Overall, IPS-fNIRS is strongest between frontal and social regions, reflecting synchronized attention, joint coordination efforts, and inferring others' actions. Asymmetrical IPS-fNIRS tends to occur between functionally similar or complementary areas, e.g., between dorsolateral PFC and MNS areas, such as the posterior superior temporal gyrus. These regions together aid the maintenance of the task goal and interpretation of others' actions and how these may suit the goal. Frontal and TPJ activity may increase during initial or novel task phases, where the team coordination style is not yet established, and may decrease as teams strengthen their SMMs and coordination.

#### 3.7.4 Breathing measures and team coordination

Two studies examined IPS-BR. As for SMMs, Filho et al. (2017) found higher IPS-BR in strongly coordinated teams in the easy, not in the challenging juggling task condition. In the challenging condition, task difficulty may have negatively impacted coordination. Thus, IPS-BR may reflect stronger team coordination. Nothing of significance was found in Henning et al. (2001).

#### 3.7.5 Electrodermal activity and team coordination

Three studies examined IPS-EDA. IPS-EDA entails that teammates' sympathetic ANS activity was temporally associated. First, Dindar et al. (2022) found that high IPS-EDA was aligned with high-coordination phases of a computer problem-solving task, indicating shared arousal and affective states among teammates. Mønster et al. (2016) found IPS-EDA to be related to perceived team tension, but not to coordinative performance. Henning et al. (2001) found no significant results. In sum, IPS-EDA reflects shared tension, arousal, or stress, which may—but do not necessarily—contribute to team coordination.

### 3.8 Multimodal integration of team coordination measures

All three multi-modal studies combined their modes into a single, unified measure. van Eijndhoven et al. (2023) incorporated HR and EDA data into a machine learning model, which successfully recalled 96% of externally rated coordination breakdowns (i.e., instances of ineffective team coordination) but exhibited low precision ( ≤ 0.29) due to a high number of false positives. This meant that the model falsely identified unrelated team dynamics as coordination breakdowns. Two studies, already discussed in Section *3.5. Multimodal Integration of SMM Measures* showed similar results for coordination. First, Stewart et al. (2021) achieved fairly good accuracy in distinguishing coordination from other aspects of problem-solving by integrating linguistic, acoustic-prosodic, facial expression, and task context features. Second, Amon et al. (2019) found that behavioral *irregularity*—measured using body movement, speech rate, and display changes—was negatively related to coordination in a visual programming task.

### 3.9 Interpersonal synchrony findings across measurement modes for team coordination

Of the 33 studies on team coordination, 23 used IPS, with the most common modes being cardiac measures (nine studies) and fNIRS (six studies). Again, diverse approaches to calculating IPS were used (see Supplementary Table 3 for details).

Studies using EEG and fNIRS reported that higher IPS was associated with stronger team coordination. In fNIRS and EEG, IPS was found in areas tied to mental state prediction, joint attention (social, temporal, frontal regions), and executive functions such as working memory and goal maintenance, consistent with prior reviews (Czeszumski et al., 2022; Réveillé et al., 2024; Wang et al., 2025).

For measures of the ANS and some bodily behaviors, such as facial expressions, the association between IPS and coordination appears to be mediated by teammates' co-varying stress and relaxation levels, or by the team's overall emotional climate (e.g., tension, trust, friendliness). Behavioral IPS, conceptualized as interpersonal regularity (e.g., in eye movements), is related to stronger coordination, whereas increased behavioral irregularity is related to weaker coordination (Amon et al., 2019).

IPS as an indicator of coordination seems to vary by task phase. It appears to rise during teamwork or challenging conditions, but not consistently during individual work. This pattern emerged in a study on IPS-EDA by Dindar et al. (2022), as well as in studies identifying high IPS as an indicator of synchronized effort during challenging conditions (HRV—Kennedy-Metz et al., 2021b; fNIRS—Xu et al., 2019).

In sum, the relationship of IPS to coordination depends on task phase, challenge level, and constraints, and on IPS calculation methods.

## 4 Discussion

In this review study, we decomposed team SA into two unobtrusively measurable components: SMMs and team coordination. We performed a systematic literature review into potential unobtrusive measures of SMMs and team coordination. In the discussion, we will overview the main findings, limitations, and practical implications.

### 4.1 Main findings

Across 52 studies, we identified and extracted measures associated with SMMs and team coordination.

The included studies strongly differed in multiple characteristics—ranging from the task environments utilized to the operationalisations of the team processes. Furthermore, the unobtrusive measures were often not the primary focus of the studies, particularly in research on team SMMs. These aspects made it difficult to compare findings. However, by bringing together findings from the various measurement modes, we could extract general patterns of behavioral and physiological activity that relate to the targeted team processes and suggest why these measures relate to the team processes. The identified patterns overlap between both team processes, which is not surprising given the theoretical co-occurrence of strong SMMs and coordination in well-performing teams (Kleinman and Serfaty, 1989; Kolbe et al., 2013).

Teams with strongly developed SMMs were characterized by *teammates predicting each other's needs and knowledge; exchanging knowledge; remaining calm;* and *attention to the task*. This was reflected across measurement modes. *Predicting needs and knowledge* concern both the ability to predict, as well as teammates' behaviors that ensure team predictability. Both were primarily reflected in cardiac and breathing rate IPS, indicative of shared mental states and similar experience of the task events, signaling a similar understanding of the task. Other evidence of this was a decrease in information requests and increased offering of unsolicited information; regular communication patterns (e.g., transitions from action- to other action-oriented statements) and regularity in eye-gaze behavior. Next, while teammates may be able to predict partner teammates' actions and movements in more structured tasks, like seen in Bourbousson et al. (2015), they may also need to monitor teammate performance (through eye-gaze direction) in high-risk tasks like naval threat scenario simulations, especially in highly challenging conditions (Espevik et al., 2011). Last, *low*-SMMs teams showed more *irregular* behavior (in body movement, speech rate, and display changes).

*Exchanging knowledge* was evident in verbal measures, including communication that focused on discussing and assigning actions, exchanging knowledge, and acknowledging teammates' statements, as well as potentially faster-paced communication to facilitate information transfer.

Regarding *remaining calm*, teams with strongly developed SMMs showed overall lowered stress levels (high HRV or low HR), or the ability to recover quickly from stress (evident through decreased HR) after highly challenging conditions; and greater executive function resources. *Heightened attention* or vigilance was reflected in EEG through increased global theta power.

Teams with strongly developed coordination can be characterized by *similar and regular teammate behavior; active social processing; remaining calm; attention, and team planning*. *Similar and regular teammate behavior* was evident in prosodic accommodation between teammates in verbal communication; high regularity in eye-gaze between teammates, and eye-gaze-to-display-changes patterns; coordinated and similar experiences of the task difficulty, especially under challenging conditions (through IPS-HR), like for SMMs; as well as high IPS-EDA, relating to shared tension, arousal, and stress; and synchronized head movements. Last, behavioral *irregularity* (in body movement, speech rate, and display changes) was related to *low-coordination* teams. *Active social processing* was primarily reflected in heightened synchronized activity in social brain regions, supporting the interpretation of teammate behavior and goals, as well as in emotional contagion expressed through shared smiling. *Remaining calm* was evidenced by high IPS-HRV. *Attention and team planning* were evident through (synchronized) brain activity in frontal regions, where cognitive effort to coordinate the planning of actions may decrease over time as teams develop SMMs; and through verbal measures, including speaking before acting and greater periods in which only one teammate was speaking.

Finally, it should be noted that some findings—e.g., ANS measures like IPS-HR—were observed only under task conditions characterized by specific (i.e. high(er)) levels of workload, stress, or difficulty. This was summarized using the word *challenging*. We interpret these findings to indicate that the task's challenge level needed to be high enough to demand strong SMMs and/or coordination, while not too high to cause a coordination breakdown.

#### 4.1.1 The interpretation of team process measures depends on task context

Some measures were inconclusive. We believe this to be a result not only of the varied operationalisations of the team processes, but also of the variation in task demands. The greatest variations were observed in verbal communication and cardiac activity.

An example in verbal communication is that while stronger SMMs were most often associated with decreased verbal frequency, some studies, examining distributed teams, showed that stronger SMMs were associated with increased verbal frequency. This seems to indicate that while teams with strong SMMs do not need to verbally communicate as much, under certain circumstances (e.g., team members being spread across locations) more verbal communication is indispensable to develop and maintain a shared understanding. A similar pattern emerged for team coordination. For team coordination, its association with *total speaking time* also differed across task environments. We reason that teams in more standardized or non-changing tasks may require less verbal communication as they develop stronger SMMs. Alternatively, teams in non-standard tasks may go through phases of less or more verbal communication as the task evolves.

Similarly, cardiac measures showed inconsistencies. Both HR and IPS-HR demonstrated positive, negative, and inconclusive relationships with team coordination across studies. This variability may partly reflect the sensitivity of HR to multiple factors such as mental stress, physical activity, and many others (Valentini and Parati, 2009). For example, in low arousing tasks, no connection between IPS-HR and team coordination was found (e.g., Mønster et al., 2016); yet in high arousing tasks, IPS-HR was found in strongly coordinated teams (e.g., Wespi et al., 2025). A possible explanation is that in low-arousal tasks, HR never increases enough to be informative of coordination. Furthermore, as discussed in the introduction, certain tasks require an optimal level of sympathetic ANS activity (Sandi, 2013). This optimal stress level may vary according to the different tasks. Consequently, the interpretation of cardiac signals may depend on both task context and the specific team process being approximated.

Taken together, these findings indicate that behavioral and physiological measures cannot be interpreted uniformly across task contexts. Their relationship with SMMs and team coordination and, ultimately, team SA, likely depends on when and how a particular process is required by the task. To use behavioral and physiological measures to estimate team SA, we need a method or approach that takes this into account and therewith produces more consistent findings (cf. *methodological research gap*; Miles, 2017). Such an approach would need to incorporate aspects of the task at hand such as whether a task is currently in a phase of team members performing independent tasks. This would satisfy findings that reported in this review that IPS-EDA was associated with team coordination specifically during task phases requiring strong coordination; and Guastello et al. (2020), who found that high IPS-EDA was positively associated with performance during phases of interdependent teamwork but negatively associated with performance during independent work. By developing more targeted, *a priori* hypotheses regarding when and how each measure should relate to a particular team process, we may advance toward a solution of the methodological gap (also argued by Waldman et al., 2019). To solve this gap, we argue that the selection and interpretation of team SA indicators should begin with an analysis of the task requirements.

#### 4.1.2 . Task analysis as the foundation for team SA measurement

Establishing a set of informative markers of team SA should begin with a task analysis to identify SA-related requirements, task constraints, and information flow (Salmon et al., 2010). *Task analysis* is defined as the study of what is required of an individual or of a team toward the execution of a given goal (Hollnagel, 2012). Task constraints shape which measuring modes and indicators can be meaningfully applied to estimate team SA. In some tasks, verbal communication is not possible, making behavior (e.g., key presses or bodily behavior) more informative. Alternatively, distributed teams may have access to less information about their teammates and the shared task environment than co-located teammates. This could be described as low team SA, *or* it could be described as the very nature of the task. A team with high team SA may accurately recognize the limitations that the task environment (i.e., distributed teams) poses and compensate through additional verbal clarifications. The question is not simply which measures can be correlated to strong team SA, but rather which measures correlate to or show that a team is engaging in the relevant and necessary team processes demanded by any particular task.

An initial task analysis can be used to define the behaviors and mental processes that are expected at different stages of a task. Then, the appropriate indicators may be chosen to detect changes in those processes and behaviors. For example, distributed teams may begin a task and employ more verbal clarifications. These may decrease after a team establishes strong SMMs, despite the physical distance between the teammates. Thus, a task analysis method may map out the expected progress of SMMs throughout the task and the elements of the task requiring active team coordination (as opposed to elements where each teammate tackles an individual task). Next, the task analysis may map out the exact sub-elements of SMMs/coordination necessary per task element. For example, a challenging phase within a certain task would require shared effort and understanding to quickly process the environment and maintain high team SA. This would be relevant to the elements of *predicting needs and knowledge* of teammates *or similar and regular behavior between teammates*, and approximated through, e.g., IPS-HR. Further, a stressful task event may elicit IPD-EDA—if all teammates involved did notice and process that such an event had occurred.

Because different aspects of team SA may manifest through different behavioral and physiological processes, these considerations also point toward the potential value of a multimodal approach to its measurement.

#### 4.1.3 Unobtrusive measurement of team SA may benefit from a multi-modal approach

Due to the complexity of team SA and the relevance of measures per task phase, we argue that a single mode cannot capture all relevant elements. Instead, team SA requires a multi-modal measurement approach. In this review, we decomposed team SA into processes expected to be more readily measurable. The results suggest that SMMs and coordination can be decomposed into yet more measurable subcomponents (section of Main Findings, in italics). This interpretation is consistent with theory indicating that processes such as SMMs are complex and encompass multiple behavioral and cognitive components (Mohammed et al., 2000; Dechurch and Mesmer-Magnus, 2010). Therefore, measuring these subcomponents requires multiple modes, each providing complementary insight into team processes.

#### 4.1.4 Interpersonal synchrony may reflect both strong team SMMs and coordination

We observed that IPS—especially in brain activity—was more frequently examined in relation to team coordination than to SMMs. Because coordination involves active and deliberate effort to understand teammates' mental state and align goals and actions, IPS may be elevated during coordinative phases. Still, IPS likely reflects a multitude of processes (Palumbo et al., 2017), and in our review, ten studies interpreted their IPS results as evidence of both team SMMs (shared understanding, goals, mental state awareness) and coordination (Filho et al., 2017; Xue et al., 2018; Xu et al., 2019; Zhang et al., 2020; Kennedy-Metz et al., 2021a; Liu et al., 2021; Sun et al., 2021; Zhang et al., 2021; Dindar et al., 2022; Wespi et al., 2025). The idea that these two processes are more intertwined than distinct has also been suggested previously (Kleinman and Serfaty, 1989; Kolbe et al., 2013). Thus, a team that has established high team SA may be characterized by high IPS, signaling both strong team SMMs and coordination (an argument also suggested by Mansikka et al., 2023) —while a team in the *process of* strengthening their SMMs may show weaker IPS (as seen in distributed teams, which are expected to have weaker SMMs; Wespi et al., 2025).

#### 4.1.5 Task-related behavioral actions are understudied as indicators of team processes

Our initial literature scans revealed no studies that used task-related behavioral actions as indicators of team processes, prompting additional scans with targeted keywords. Still, no relevant studies were found. Two studies examined display changes alongside other factors, like eye-gaze (Amon et al., 2019; Vrzakova et al., 2019), but did not use actions like key presses as primary drivers of the display changes. Such interactions with the display may nonetheless reflect coordinative actions and shared knowledge and can be extracted through an initial task analysis to fit each task environment. Therefore, we recommend further study of how task-related behavioral actions can be measured and used for the analysis of team processes and team SA.

### 4.2 Limitations

The search strategy we used has its limitations. First, while we included synonyms of the targeted processes (SMM and team coordination) as keywords for our search, we may have missed studies which had a different scope. For example, we could have missed studies that may have indirectly addressed our targeted team processes as intrinsic aspects of team performance. Yet, our goal was to remain focused on our target constructs. We included synonyms of the team processes to cover more literature. Second, because of practical limitations, only a single reviewer (EZ) performed the screening and selection of literature, as well as the information extraction. Although this is a deviation from the PRISMA protocol, we expect it to have little effect because edge cases were discussed with the full research team. Third, we only examined studies that were published in the year 2000 and later. Reasons to exclude earlier work were 1) wanting to focus on novel technologies, and 2) the oldest reference included in the outstanding review paper on SA by Zhang et al. (2023) was from 2001 (Merchant et al., 2001).

Another limitation, referring to the outcome of the review, is that the included studies used highly diverse approaches to estimating the strength of SMMs and team coordination, making direct comparisons between studies difficult. As discussed in the following section on future work, the reviewed measures should be investigated more systematically in relation to team SA.

### 4.3 Future work

Based on the above discussion, we recommend future work in the following direction.

First, the task analysis approach should be developed and tested as the basis of selecting unobtrusive measures of team SA. Rather than selecting measures ad hoc, future work may investigate the characteristics of any given task and determine which measures would theoretically be most informative. In this way, guidelines may be established for the selection and interpretation of these measures across tasks and contexts. These guidelines can be validated by examining whether the theoretically justified predictions were accurate about the resulting unobtrusive measurements.

Next, these predictions of team SA should be validated against an established and independent measurement or manipulation of team SA. For example, the SAGAT (Endsley, 2020) can be used as a measure, or in a study where teammates have visual information about each other's current task, this visual channel may be experimentally interrupted. This would be a priori expected to weaken team SA—participants can no longer see what their teammate is doing. A task analysis may be performed to develop exact expectations about how the verbal behavior of participants will change. Researchers could then examine whether these hypothesized changes in verbal behavior explain variance in independently measured team SA. Such an approach would provide a stronger empirical basis for the proposed relation between unobtrusive measures and team SA. Additionally, it would further corroborate the framework by Salmon et al. (2008). More broadly, research in the direction of utilizing a task analysis as the first step to team SA measurement could advance the field beyond the ad hoc selection of unobtrusive measures.

### 4.4 Practical implications

In this paper, we propose an approach for unobtrusively measuring team SA by identifying indicators that can estimate the team processes involved (Salmon et al., 2008). We propose that these efforts begin with a task analysis; and that the interpretation of the measures will depend on the exact task environment and team processes progression. We identified further sub-parts of these team processes (e.g., *remaining calm, heightened attention*, etc.). These indicators could eventually be automated for real-time application, for example, in team training, which is a crucial component of team SA (Meng et al., 2022). A continuous, unobtrusive and automatic measure of team SA could support the teaching staff, reduce training time, and improve error prevention. For example, for certain contexts, teams may be instructed to utilize closed-loop communication by affirming they have heard a teammate's instruction whereas in other contexts biofeedback may be used to inform the team on IPS, which may improve teammate awareness. Although the optimal combination and application of these measures remain open questions for future projects, this paper proposes an initial selection of measures and a task analysis approach.

## 5 Conclusion

In this systematic review, we decomposed team SA into team processes that are likely to be unobtrusively measurable. To this end, we adapted Salmon et al. (2008)'s framework of team SA and focused specifically on the team processes of SMMs and coordination. In the 19 selected studies on unobtrusively measuring SMMs and 33 on team coordination, measures of various modes were used, with cardiac and verbal measures being most used to estimate team SMMs, and fNIRS and cardiac measures used for team coordination. IPS (in various modes) was a popular measure for team coordination. We found that task-related behavioral actions, like key presses, are understudied as unobtrusive measures. Furthermore, we found that SMMs and coordination are broken down into further subcomponents, all measured through different but complementary modes. A multi-modal measurement approach may result in the most meaningful estimate, but it will still depend on the task and context. Therefore, we recommend performing a task analysis to determine how the measurable processes underlying team SA develop over the course of a task and, given task constraints, select appropriate modes and measures from those identified in this review.

## Data Availability

The original contributions presented in the study are included in the article/Supplementary material, further inquiries can be directed to the corresponding authors.
