# Supplementary material for: Review on unobtrusive estimation of team processes underlying team situation awareness: shared mental models and team coordination
Source: Front Neuroergon. 2026 Oct 14;7:1864518. doi: 10.3389/fnrgo.2026.1864518 (PMC13713447; doi:10.3389/fnrgo.2026.1864518)
Supplement: Supplementary file 1 [file Data_Sheet_1.docx]

Supplementary Material

# 1 Supplementary Data

## 1.1 Supplementary Tables

**Supplementary Table 1**
*A brief overview of all included papers in the review.*

| **Team process(es)** | **Measurement mode(s)** | | **Type(s) of measure(s)** | **Authorship (APA)** |
| --- | --- | --- | --- | --- |
| Shared mental models | Behaviour | Verbal communication | | Blaser and Seiler (2019); Badke-Schaub et al. (2011); Bönsel and Müller (2021); Eldadi and Tenenbaum (2025); Lausic et al. (2009); Mamykina et al. (2016) |
|  |  | Bodily behaviour | | Bourbousson et al. (2015) |
|  | Behaviour; Physiology | Verbal communication; cardiac measures | | Espevik et al. (2006) |
|  |  | Verbal communication; eye-tracking; cardiac measures | | Espevik et al. (2011) |
|  | Physiology | Cardiac measures | | Del Rosario et al. (2024); Sætrevik (2015): Study 2 |
|  |  | Cardiac measures; Electroencephalography | | Hoyle et al. (2020): Study 3 |
| Shared mental models; team coordination | Behaviour | Verbal communication | | Pugh et al. (2022) |
|  |  | Eye-tracking | | Vrzakova et al. (2019) |
|  | Physiology | Cardiac measures | | Kennedy-Metz et al. (2021a); Kennedy-Metz et al. (2021b) |
|  |  | Cardiac measures; breathing | | Filho et al. (2017) |
|  | Multiple modes of measurement | Verbal communication; bodily behaviour; display interactions | | Amon et al. (2019) |
|  |  | Verbal communication; bodily behaviour; task information features | | Stewart et al. (2021) |
| Team coordination | Behaviour | Verbal communication | | De Looze et al. (2015); Feese et al. (2013); Hoyle et al. (2020): Study 1 |
|  | Behaviour; Physiology | Bodily behaviour; cardiac measures | | Strang et al. (2014) |
|  |  | Bodily behaviour; Electrodermal activity | | Mønster et al. (2016) |
|  | Physiology | Cardiac measures | | Fusaroli et al. (2015): Study 1; Fusaroli et al. (2015): Study 2; Henning et al. (2001)^a^; Powezka et al. (2022); Veerabhadrappa et al. (2021); Wespi et al. (2025); |
|  |  | Electroencephalography | | Astolfi et al. (2010); Dodel et al. (2011); Dodel et al. (2020); Reinero et al. (2021); Zamm et al. (2021) |
|  |  | Functional near-infrared spectroscopy | | Hayne et al. (2023); Li et al. (2020); Liu et al. (2021); Mayseless et al. (2019); Sun et al. (2021); Xu et al. (2019); Xue et al. (2018); Zhang et al. (2020); Zhang et al. (2021) |
|  |  | Electrodermal activity | | Dindar et al. (2022); |
|  | Multiple modes of measurement | Cardiac measures; Electrodermal activity | | van Eijndhoven et al. (2023) |

*Notes.* ^a^ marked non-significant results. A total of 52 papers were included in the systematic review – 19 papers on shared mental models, and 33 papers on team coordination. Abbreviations: APA, American Psychological Association.

**Supplementary Table 2**
*An Overview of the Papers Included in the Qualitative Synthesis regarding Unobtrusive Measures of Team Shared Mental Models*

| **#** | **Authorship (APA)** | ***N; n*** | **Task** | **Operationalisation of SMMs** | **Measure** | **Result** |
| --- | --- | --- | --- | --- | --- | --- |
| **Verbal Communication** | | | | | | |
| 1 | Badke-Schaub et al. (2011) | -; 7 | Product design problem. | Time on task. | • Communication frequency (% of total communication) per communication content type (manually coded with pre-defined categories). | Decreased planning statements. |
| 2 | Blaser and Seiler (2019) | 46; 2 | Real-life football. | Self-report questionnaire. | • Communication frequency (frequency of meaningful communication  units per measuring point). | Decreased communication frequency. |
|  |  |  |  |  | • Communication frequency (% of total communication) per communication content type (manually coded with pre-defined categories). | Decreased task-relevant statements. |
| 3 | Bönsel and Müller (2021) | 60; 2 | Cooperative fault diagnosis. | Experimentally controlled. | • Communication distribution. | Similar communication distribution between team members. |
|  |  |  |  |  | • Communication frequency (total number of statements), specifically for communication content types (manually coded with pre-defined categories). | Increased knowledge exchange statements. |
|  |  |  |  |  |  | Decreased task-relevant statements. |
|  |  |  |  |  | • Communication frequency (total number of statements). | Decreased communication frequency. |
| 4 | Eldadi and Tenenbaum (2025) | 40; 5 | Online gaming^V^ (*Counter-strike*). | Participant experience. | • Communication pace (word count per second). | Quicker (concise) statements, overall higher word count and statements during the task. |
|  |  |  |  |  | • Communication patterns (content transitions through sequential content analysis). | Increased frequency of transitions from action-oriented to action-oriented statements, emotional/motivational, acknowledgement, and knowledge exchange statements; uncertainty to acknowledgement statements; and knowledge exchange to action-oriented, acknowledgement, or knowledge exchange statements. |
|  |  |  |  |  | • Communication frequency (proportion of talk time relative to total task time). | Increased communication frequency. |
|  |  |  |  |  | • Communication frequency (proportion of available time utilised for communication), specifically for communication content types (manually coded with pre-defined categories). | Increased communication frequency, specifically – increased action-oriented, motivational/emotional, knowledge exchange, acknowledgement, and uncertainty statements. |
|  |  |  |  |  |  | Decreased task-irrelevant statements. |
| 5 | Espevik et al. (2006) | 24; 6 | Simulated tactical submarine scenario. | Participant experience + self-report questionnaire. | • Communication strategy (amount of information requests, manually coded with pre-defined categories). | Decreased information requests. |
|  |  |  |  |  | • Communication frequency (number of verbalisations per minute). | Decreased communication frequency. |
| 6 | Espevik et al. (2011) | 28; 3 | Simulated naval threat scenario. | Participant experience. | • Communication frequency (% of total communication) per communication content type (manually coded with pre-defined categories). | Decreased task-irrelevant communication in co-located teams. |
|  |  |  |  |  |  | Increased knowledge exchange statements in physically distributed teams. |
|  |  |  |  |  | • Communication strategy (amount of information requests, manually coded with pre-defined categories). | Increase in global anticipation ratio (number of transfers, divided by number of requests for information), especially during challenging conditions, compared to easy conditions. |
|  |  |  |  |  | • Communication frequency (statements per minute). | Increased communication frequency, especially during challenging conditions, compared to easy conditions (in physically distributed teams). |
| 7 | Lausic et al. (2009) | 10; 2 | Real-life doubles tennis. | Performance-associated. | • Communication patterns (transitions from one content category to another; content was manually coded with pre-defined categories). | Increased transitions from action-oriented to action-oriented or acknowledgement; acknowledgement to action; knowledge exchange to acknowledgement or knowledge exchange; emotional/motivational to uncertainty, action, knowledge exchange, or emotional/motivational; and uncertainty to acknowledgement statements. |
| 8 | Mamykina et al. (2016) | -; - | Real-life patient information handoff. | Associated with a task phase (high SMMs during information handoff). | • Communication content overlap (content coded through a cluster analysis. Overlap assessed through a modified Pyramid method for linguistic and semantic similarity). | High congruency in the patient representations between different team members. |
| 9 | Pugh et al. (2022) | 285; 3 | Physics and programming games^V^. | Subjective assessment by experts. | • Language-based models (BERT and LIWC). | High model accuracy (AUROC = .80) in distinguishing the shared knowledge facet from others (*coordinating and maintaining team function*). |
| **Eye-Tracking** | | | | | | |
| 6 | Espevik et al. (2011) | 28; 3 | Simulated naval threat scenario^V^. | Participant experience. | • Number of glances towards team member or their task/actions. | The number of eye glances towards teammate actions or needs on a digital display remained the same in both easy and challenging conditions. |
| 10 | Vrzakova et al. (2019) | 57; 3 | Visual computer programming task^V^. | Subjective assessment by experts. | • **IPS:** Regularity in individual-level eye-gaze (assessed through eye tracker) to display change (assessed through video recordings of the screen activity – pixel changes), analysed through cross-recurrence quantification. | High regularity between individual-level eye-gaze towards the display and display changes. |
| **Bodily Behaviour** | | | | | | |
| 11 | Bourbousson et al. (2015) | 10; 5 | Real-life basketball. | Participant experience. | • Social network analysis metrics - network density, outdegree of each node, and reciprocity of connections. Team members represented nodes, and team members’ awareness of other members (assessed through interviews and video recordings of performance) represented node relations. | Low overt awareness of teammates (evident through low network density, low outdegree of each teammate, and low reciprocity values). |
| **Cardiac Measures** | | | | | | |
| 12 | Del Rosario et al. (2024) | 26; - | Real-life surgery. | Associated with a task phase (similar affective states were assumed during high workload task periods). | • **IPS:** Correlation analysis in HR data between teammates. | Positively correlated HR between teammates. |
| 5 | Espevik et al. (2006) | 24; 6 | Simulated tactical submarine scenario. | Participant experience + self-report questionnaire. | • HR changes (beats per minute). | Equal HR during easy and challenging conditions. |
| 6 | Espevik et al. (2011) | 28; 3 | Simulated naval threat scenario^V^. | Participant experience. | • HR changes (beats per minute). | Equal HR during easy and challenging conditions. |
|  |  |  |  |  |  | Recovery in HR after challenging conditions. |
| 13 | Filho et al. (2017) | 2; 2 | Controlled juggling task. | Participant experience + experimentally controlled. | • **IPS:** Correlation analysis in HR data between teammates. | Positively correlated HR between teammates (in both easy and challenging conditions). |
| 14 | Hoyle et al. (2020): Study 3 | 24; 2 | Football simulation online gaming^V^. | Time on task. | • HRV changes. | Increased HRV. |
| 15 | Kennedy-Metz et al. (2021a) | 9; 9 | Real-life surgery. | Associated with a task phase (high workload surgery phases require high SMMs). | • Average HRV (measured via RMSSD) between teammates. | No statistically significant difference between the average teammate HRV. |
| 16 | Kennedy-Metz et al. (2021b) | 15; - | Real-life surgery. | Associated with a task phase (high workload surgery phases require high SMMs). | • **IPS:** Synchronised peaks in HRV (measured via RMSSD and identified through manual observation) across team members. | Increased frequency of synchronised negative peaks in HRV, especially during high SMM task phases. |
| 17 | Sætrevik (2015): Study 2 | 45; 9 | Simulated emergency response teaming. | Self-report questionnaire. | • HRV changes (measured through RMSSD). | Increased HRV. |
| **Electroencephalography** | | | | | | |
| 14 | Hoyle et al. (2020): Study 3 | 24; 2 | Football simulation online gaming^V^. | Time on task. | • Theta power (21-channel EEG). | Increased absolute theta power in frontal channels. |
| **Breathing** | | | | | | |
| 13 | Filho et al. (2017) | 2; 2 | Controlled juggling task. | Participant experience + experimentally controlled. | • **IPS:** Correlation analysis of breathing rate between teammates. | Positively correlated breathing rate between teammates (only in easy, not in the challenging condition). |
| **Multiple Modes of Measurement** | | | | | | |
| 18 | Amon et al. (2019) | 96; 3 | Visual computer programming task^V^. | Subjective assessment by experts. | • **IPS:** Team-level behavioural regularity (computed through multidimensional RQA based on body movement, speech rate, and display interactions into one metric). | Behavioural irregularity related to low team SMMs. |
| 19 | Stewart et al. (2021) | 37; 3 | Visual programming task^V^. | Subjective assessment by experts. | • Multimodal Random Forest models involving acoustic-prosodic, linguistic (through automated speech recognition), facial, body movement (video recordings), and task information features. | Fairly good model accuracy (AUROC = .75) in distinguishing the shared knowledge facet from others (*negotiation/coordination* and *maintaining team functions*). |

*Notes.* ^V^ marked teaming in virtual environments. Type of measure is indicated as the division heading (e.g., *verbal communication*). The final analysis included 19 papers. All results correspond to improved team SMMs, unless stated otherwise (e.g., Amon et al., 2019). Abbreviations: APA, American Psychological Association; N, total sample size of paper; n, team size in the paper; SMMs, shared mental models; BERT, deep transfer learning language model; LIWC, Linguistic Inquiry Word Count dictionary, language model; AUROC, area under the receiver operating characteristic curve, the ratio between true and false positive rates; IPS, interpersonal synchrony; HR, heart rate; HRV, heart rate variability; RMSSD, root mean square of successive differences; EEG, electroencephalography; RQA, recurrence quantification analysis.

**Supplementary Table 3**
*An Overview of the Papers Included in the Qualitative Synthesis regarding Unobtrusive Measures of Team Coordination*

| **#** | **Authorship (APA)** | ***N;* *n*** | **Task** | **Operationalisation of Team Coordination** | **Measure** | **Result** |
| --- | --- | --- | --- | --- | --- | --- |
| **Verbal Communication** | | | | | | |
| 1 | De Looze et al. (2015) | 12; 2 | Crew resource management^V^. | Subjective assessment by experts (evaluated a *total coordination mean*, composed of multiple, correlated metrics, including adaptability, communication, etc.). | • Prosodic accommodation (calculated per speaker using prosodic features – pitch, energy, duration of pauses, etc. Features were correlated between speakers). | High per-speaker prosodic accommodation. |
|  |  |  |  |  | • Simultaneous speech (teammates speaking at the same time. Verbal data was automatically annotated for speaking and silence units). | More simultaneous speech. |
| 2 | Feese et al. (2013) | 10; 5 | Real-life firefighting training. | Subjective assessment by experts. | • Speech activity level (calculated every second as the time fraction of a two-second-long window in which voice activity was detected through a voice activity detection algorithm) and behavioural action. | Team, or team leaders, communicated a plan before action execution, not during. |
| 3 | Hoyle et al. (2020): Study 1 | 48; 2 | Football simulation online gaming^V^. | Time on task. | • Simultaneous speech (teammates speaking at the same time, derived through a sociometric badge). | Less simultaneous speech. |
|  |  |  |  |  | • Amount of listening (single teammate speaking at a time, derived through a sociometric badge). | Increased amount of focused listening. |
|  |  |  |  |  | • Total speaking time (silence, compared to speaking, derived through a sociometric badge). | Decreased total silence time. |
| 4 | Pugh et al. (2022) | 285; 3 | Physics and programming games^V^. | Subjective assessment by experts. | • Language-based models (BERT and LIWC). | High model accuracy (AUROC = .80) in distinguishing the coordination facet from others *(shared knowledge* and *maintaining team function*). |
| **Eye-tracking** | | | | | | |
| 5 | Vrzakova et al. (2019) | 57; 3 | Visual computer programming task^V^. | Self-report questionnaire (mean of three self-rated items: *communication, cooperation, and agreeableness*, due to high intercorrelation). | • **IPS:** System complexity – how often the same system was revisited (entropy in the diagonal line lengths from cross RQA) in the relation between individual-level eye-gaze (assessed through eye tracker) to display changes (assessed through video recordings of the screen activity – pixel changes), analysed through cross RQA. | High system complexity in the relation between individual-level eye-gaze and display changes is negatively related to team coordination. |
|  |  |  |  | Subjective assessment by experts. | • **IPS:** System complexity – how often the same system was revisited (entropy in the diagonal line lengths from cross RQA) in the relation between individual-level eye-gaze (assessed through eye tracker) to display changes (assessed through video recordings of the screen activity – pixel changes). | High system complexity in the relation between individual-level eye-gaze and display changes. |
|  |  |  |  |  | • **IPS**: Regularity in team-level eye-gaze (assessed through eye tracker) to display changes (assessed through video recordings of the screen activity – pixel changes), analysed through multidimensional RQA. | High regularity between team-level eye-gaze towards the display and display changes. |
|  |  |  |  |  | • **IPS:** Synchrony in eye-gaze (assessed through eye tracker) of all teammates and its correspondence to the display area of interest changes (assessed through video recordings of the screen activity – pixel changes), analysed through multidimensional RQA. | High synchrony in the eye-gaze of all teammates and its correspondence to the active display area of interest. |
|  |  |  |  |  | • **IPS:** Synchrony in eye-gaze (assessed through eye tracker) of all teammates (analysed through multidimensional RQA). | High synchrony in the eye-gaze of all teammates |
| **Bodily Behaviour** | | | | | | |
| 6 | Strang et al. (2014) | 80; 2 | Puzzle task^V^. | Teaming conditions. | • **IPS:** Cross-fuzzy entropy, cross-correlation, and cross RQA in head postural sway movements between teammates. | High head postural sway movement IPS (only in cross RQA) in real teams versus an artificial team, made up of individuals performing the task. |
| 7 | Mønster et al. (2016) | 153; 3 | Assembly line creation task. | Teaming conditions + self-report questionnaire. | • **IPS:** Cross RQA in zygomaticus electromyographical activity between teammates. | High IPS in zygomaticus electromyographical activity related to emotional aspects of coordination. |
| **Cardiac Measures** | | | | | | |
| 8 | Filho et al. (2017) | 2; 2 | Controlled juggling task. | Participant experience + experimentally controlled. | • **IPS:** Correlation analysis in HR data between teammates. | Positively correlated HR between teammates (in both easy and challenging conditions). |
| 9 | Fusaroli et al. (2015): Study 1 | 29; 4-5 | Creative construction task. | Teaming conditions (real vs false-matched teams; and individual vs collective task phases). | • **IPS:** Cross RQA in HR between teammates: assessing level (recurrence rate) and stability (length of sequences repeated across time-series) of HR IPS between teammates. | High stability in HR IPS in real teams across both collective and individual (team) task phases. |
|  |  |  |  |  |  | Increasing HR IPS over time in real teams in collective tasks. |
|  | Fusaroli et al. (2015): Study 2 |  |  | Performance-associated (behavioural and speech coordination). |  | High and more stable (especially with improved speech coordination) HR IPS. |
| 10 | Kennedy-Metz et al. (2021a) | 9; 9 | Real-life surgery. | Associated with a task phase (high workload surgery phases require high SMMs). | • Average HRV (measured via RMSSD) between teammates. | No statistically significant difference between the average teammate HRV. |
| 11 | Kennedy-Metz et al. (2021b) | 15; - | Real-life surgery. | Associated with a task phase (high workload surgery phases require high SMMs). | • **IPS:** Synchronised peaks in HRV (measured via RMSSD and identified through manual observation) across team members. | Increased frequency of synchronised negative peaks in HRV, especially during high SMM task phases. |
| 12 | Powezka et al. (2022) | 12; 2 | Real-life surgery. | Performance-associated. | • **IPS:** Phase synchrony index in HRV between teammates. HRV metrics used: number of IPS trends, area under IPS trend curve, length of PS trends, and number of IPS trends – all per hour of procedure. | High HRV IPS. |
| 13 | Strang et al. (2014) | 80; 2 | Puzzle task^V^. | Teaming conditions. | • **IPS:** Cross-fuzzy entropy, cross-correlation, and cross RQA in HR between teammates. | High HR IPS (cross-fuzzy entropy and cross RQA) in real teams versus an artificial team, made up of individuals performing the task. |
|  |  |  |  |  |  | HR IPS in real versus false-matched teams (a member of one real team artificially matched with a member from another real team) was only higher compared to the false-matched teams of teammates holding the same team role. |
| 14 | Veerabhadrappa et al. (2021) | 38; 2 | Programming task. | Teaming conditions. | • **IPS:** Pearson’s correlation analysis in HRV. HRV was calculated using a linear (SDNN of inter-beat interval) and a non-linear method (RQA metrics). | High HRV IPS (in both SDNN and RQA-based metrics of HRV). |
| 15 | Wespi et al. (2025) | 208; 2 | Medical training simulation^V^. | Teaming conditions. | • **IPS:** Dynamic time warping algorithm in HR and HRV (calculated as RMSSD or SDNN) between teammates. | High HR IPS. |
|  |  |  |  |  |  | Low HRV (RMSSD) IPS. |
|  |  |  |  |  |  | High HRV (SDNN). |
|  |  |  |  | Teaming conditions + time on task. |  | Higher HRV (SDNN) IPS in the second high-coordination task, compared to the first. |
|  |  |  |  |  |  | Lower HR IPS in the second high-coordination task, compared to the first. |
| **Electroencephalography** | | | | | | |
| 16 | Astolfi et al. (2010) | 28; 2 | Real-life card game. | Teaming conditions. | • **IPS:** Partial Directed Coherence (64-channel EEG). | High IPS in the dorsolateral pre-frontal areas of the teammate leader, with the activity in the anterior cingulate cortex in the teammate follower in the alpha, beta, and gamma bands. |
| 17 | Dodel et al. (2011) | 8; 2 | Simulated combat scenario^V^. | Teaming conditions. | • **IPS:** Subspace of joint brain dynamics (through Singular Value Decomposition and team manifold approximation; 256-channel EEG). | Three-dimensional, dominant team subspace (IPS) in the right PFC. |
| 18 | Dodel et al. (2020) | 32; 2 | Virtual room clearing task^V^. | Performance-associated. | • **IPS:** Phase locking value and joint wavelet power patterns (60-channel EEG). | Low leader beta synchronised with high follower beta power, and low leader gamma power with low follower beta power (in beta and gamma rhythms). Both effects were observed at medial centro-parietal electrode sites. |
| 19 | Reinero et al. (2021) | 174; 4 | Problem-solving task. | Teaming conditions. | • **IPS:** Their own pairwise synchrony equation (14-channel EEG). | Smaller decrease in EEG IPS over time on task. |
| 20 | Zamm et al. (2021) | 40; 2 | Piano playing. | Teaming conditions. Examined *temporal coordination* – synchrony of actions between individuals in rhythmic activities like musical performance. | • **IPS:** Amplitude Envelope Correlation (24-channel EEG). | Higher IPS in oscillatory amplitude fluctuations at the performance frequency. |
| **Functional Near-Infrared Spectroscopy** | | | | | | |
| 21 | Hayne et al. (2023) | 84; 2 | Computer Balloon Analogue Risk Task (BART) ^V^. | Teaming conditions. | • PMC activation (42-channel fNIRS). | Decreased PMC activity. |
|  |  |  |  |  | • Bilateral dorsolateral PFC (42-channel fNIRS). | Decreased bilateral dorsolateral PFC activity. |
| 22 | Li et al. (2020) | 48; 2 | Joint drawing task^V^. | Performance-associated + teaming conditions. | • **IPS:** Wavelet Transform Coherence (22-channel fNIRS). | High dorsolateral PFC (channel 15) IPS. |
| 23 | Liu et al. (2021) | 180; 9 | Real-life drumming task. | Teaming conditions. | • Left TPJ Global Network efficiency (30 and 40 channel fNIRS. Network efficiency calculated using network analysis and Graph theory). | Higher average left TPJ Global Network Efficiency. |
|  |  |  |  |  | • Medial PFC global and local network efficiency (30 and 40 channel fNIRS. Network efficiency calculated using network analysis and Graph theory). | Higher average medial PFC Global and Local Network Efficiency. |
|  |  |  |  |  | • **IPS:** Cross-correlation in fNIRS between teammates (32- and 40-channel fNIRS). | High IPS in medial PFC and left TPJ. |
| 24 | Mayseless et al. (2019) | 56; 2 | Creative design task. | Teaming conditions. | • **IPS:** Wavelet Transform Coherence in fNIRS between teammates (22-channel fNIRS). | High IPS between anterior PFC and posterior STG; inferior temporal gyrus and posterior STG. |
|  |  |  |  | Self-report questionnaire. |  | High IPS in anterior PFC to posterior STG. |
|  |  |  |  | Subjective assessment by experts. |  | Over time, IPS between the anterior PFC and TPJ decreased (only during the creative task), coinciding with increased coordination. |
| 25 | Sun et al. (2021) | 86; 2 | Joint drawing task^V^. | Performance-associated + teaming conditions. | • **IPS:** Wavelet Transform Coherence in fNIRS between teammates (22-channel fNIRS). | High IPS in the left frontopolar region. |
| 26 | Xu et al. (2019) | 33; - | Crisis event management virtual reality task^V^. | Teaming conditions. | • Anterior PFC activation (1-channel fNIRS) | Increase in HbO towards the end of the coordinative task |
|  |  |  |  |  | • **IPS:** Wavelet Transform Coherence (1-channel fNIRS – anterior PFC). | High IPS-HbR (although this was the case during periods of shared information processing and no coordination). |
|  |  |  |  |  |  | High IPS-HbO. |
|  |  |  |  |  |  | Increasing IPS-HbO/HbR (especially in increasingly challenging conditions). |
| 27 | Xue et al. (2018) | 90; 2 | Realistic creativity problem. | Subjective assessment by experts. | • **IPS:** Wavelet Transform Coherence in fNIRS between teammates (22- and 24-channel fNIRS). | High IPS in the right dorsolateral PFC. |
|  |  |  |  |  |  | High IPS in right TPJ. |
| 28 | Zhang et al. (2020) | 62; 2 | Moral dilemma game. | Teaming conditions + experimentally controlled. | • **IPS:** Wavelet Transform Coherence (30-channel fNIRS). | High IPS in the right IFG. |
| 29 | Zhang et al. (2021) | 62; 2 | Moral dilemma game. | Experimentally controlled. | • Dorso-lateral PFC activity (22-channel fNIRS). | High dorsolateral PFC activity. |
|  |  |  |  |  | • Middle frontopolar area activity (22-channel fNIRS). | High middle frontopolar area activity. |
|  |  |  |  |  | • **IPS:** Wavelet Transform Coherence in fNIRS between teammates (22-channel fNIRS). | High IPS in the right IFG. |
| **Breathing** | | | | | | |
| 8 | Filho et al. (2017) | 2; 2 | Controlled juggling task. | Participant experience + experimentally controlled. | • **IPS:** Correlation analysis in breathing rate data between teammates. | Positively correlated breathing rate between team members in easy conditions, not in challenging conditions. |
| **Electrodermal Activity** | | | | | | |
| 29 | Dindar et al. (2022) | 77; 2-3 | Computer-based problem solving. | Teaming conditions + subjective assessment by experts. | • **IPS:** Synchronised peaks in EDA between teammates. | High IPS in EDA peaks between teammates, corresponding to high-coordination task phases. |
| 7 | Mønster et al. (2016) | 153; 3 | Assembly line creation task. | Teaming conditions + self-report questionnaire. | • **IPS:** Cross RQA in EDA data between teammates. | High EDA IPS related to team tension (not performance-related coordination aspects). |
| **Multiple Modes of Measurement** | | | | | | |
| 30 | Amon et al. (2019) | 96; 3 | Visual computer programming task^V^. | Subjective assessment by experts. | • **IPS:** Team-level behavioural regularity (computed through multidimensional RQA based on body movement, speech rate, and display interactions into one metric). | Behavioural irregularity related to low team coordination. |
| 31 | Stewart et al. (2021) | 37; 3 | Visual programming task^V^. | Subjective assessment by experts. | • Multimodal Random Forest models involving acoustic-prosodic, linguistic (through automated speech recognition), facial, body movement (video recordings), and task information features. | Good model accuracy (AUROC = .67) in distinguishing the coordination facet from others (*shared knowledge* and *maintaining team functions*). |
| 32 | van Eijndhoven et al. (2023) | 47; 4 | Simulated crisis game. | Subjective assessment by experts – measured *coordination breakdowns*. | • HR based on windowed multidimensional RQA and EDA based on windowed synchronisation coefficient analysis combined into a machine learning model. | Machine learning model recalled 96% of the externally rated coordination breakdowns, but precision scores were low (≤.29). |

*Notes.* ^V^ marked teaming in virtual environments. Type of measure is indicated as a division heading (e.g., *verbal communication*). The final analysis included 33 papers – Henning et al. (2001) is not included in this table, due to non-significant findings. All results correspond to improved team coordination, unless stated otherwise (e.g., Amon et al., 2019). Abbreviations: APA, American Psychological Association; N, total sample size of paper; n, team size in the paper; BERT, deep transfer learning language model; LIWC, Linguistic Inquiry Word Count dictionary, language model; AUROC, area under the receiver operating characteristic curve, the ratio between true and false positive rates; IPS, interpersonal synchrony; RQA, recurrence quantification analysis; HR, heart rate; HRV, heart rate variability; RMSSD, root mean square of successive differences; SDNN, standard deviation of normal-normal; EEG, electroencephalography; PFC, pre-frontal cortex; PMC, primary motor cortex; fNIRS, functional near-infrared spectroscopy; TPJ, temporo-parietal junction; STG, superior temporal gyrus; HbO, oxygenated hemoglobin; HbR, de-oxygenated hemoglobin; IFG, inferior frontal gyrus; EDA, electrodermal activity.
